# A ratiometric-based measure of gene co-expression

**DOI:** 10.1186/1471-2105-15-331

**Published:** 2014-11-20

**Authors:** Anna CT Abelin, Georgi K Marinov, Brian A Williams, Kenneth McCue, Barbara J Wold

**Affiliations:** Division of Biology and Biological Engineering, California Institute of Technology, 1200 East California Blvd, Pasadena, CA 91125 USA

**Keywords:** Gene expression analysis, Ratiometric analysis, Transcriptome analysis, RNA-seq, Single-cell RNA-seq, Mutual information, Pearson correlation, RA

## Abstract

**Background:**

Gene co-expression analysis has previously been based on measures that include correlation coefficients and mutual information, as well as newcomers such as MIC. These measures depend primarily on the degree of association between the RNA levels of two genes and to a lesser extent on their variability. They focus on the similarity of expression value trajectories that change in like manner across samples. However there are relationships of biological interest for which these classical measures are expected to be insensitive. These include genes whose expression levels are ratiometrically stable and genes whose variance is tightly constrained. Large-scale studies of relatively homogeneous samples, including single cell RNA-seq, are experimental settings in which such relationships might be especially pertinent.

**Results:**

We develop and implement a ratiometric approach for detecting gene associations (abbreviated RA). It is based on the coefficient of variation of the measured expression ratio of each pair of genes. We apply it to a collection of lymphoblastoid RNA-seq data from the 1000 Genomes Project Consortium, a typical sample set with high overall homogeneity. RA is a selective method, reporting in this case ~1/4 of all possible gene pairs, yet these relationships include a distilled picture of biological relationships previously found by other methods. In addition, RA reveals expression relationships that are not detected by traditional correlation and mutual information methods. We also analyze data from individual lymphoblastoid cells and show that desirable properties of the RA method extend to single-cell RNA-seq.

**Conclusion:**

We show that our ratiometric method identifies biologically significant relationships that are often missed or low-ranked by conventional association-based methods when applied to a relatively homogenous dataset. The results open new questions about the regulatory mechanisms that produce strong RA relationships. RA is scalable and potentially well suited for the analysis of thousands of bulk-RNA or single-cell transcriptomes.

**Electronic supplementary material:**

The online version of this article (doi:10.1186/1471-2105-15-331) contains supplementary material, which is available to authorized users.

## Background

Analyses of gene co-expression that use measures of association, such as the Pearson and Spearman correlation coefficients, the squared Pearson correlation coefficient (R^2^), and mutual information, are ubiquitous in modern biology. These measures of association are the basis for the most widely used clustering techniques [1], and are also used for a diversity of network motif and inference algorithms ( [2–4] and references therein).

They effectively highlight relationships between genes whose expression levels co-vary across a set of heterogeneous samples, and the results they produce are quite similar [5]. And while newer approaches, such as the maximum information coefficient (MIC) [3], have been presented, their novelty relative to existing measures has been disputed [6].

The properties and similarities among current methods raise two related issues. First, there is a rapidly growing repertoire of transcriptome studies that each consist of large numbers of homogeneous samples, including single-cell RNA-seq studies. Is there an effective way to detect biological relationships from these expression data that will not depend heavily on sample heterogeneity? Second, are there classes of relationships of potential biological importance that have been persistently missed or undervalued by the existing methods?

To address these questions we sought a measure that would detect previously missed relationships, while including known ones. Others have pointed to limitations in the standard methods, although they are widely unappreciated (see [7]). Relevant to the current study is the fact that variability affects the usual measures of gene co-expression in sometimes unexpected ways, including causing some genes to score as not co-expressed, even though scrutiny of the evidence suggests that they are.

In this study we develop and implement a conceptually different approach, termed the ratiometric method (RA), which ranks each gene pair *A* and *B* based on the stability of the ratios of their expression values *A/B* and *B/A* across samples. The more stable the ratio is, the more strongly the relationship will score. From a biological point of view, the objective is to be sensitive to gene pair relationships in which the relative expression levels are constant, irrespective of whether the absolute levels differ widely. Further, we are interested in finding and scoring expression patterns that are highly constrained within and between the components of different cellular pathways and structures. These hypothesized tight quantitative relationships at the RNA level are of interest, because they may reflect novel regulatory strategies and constraints. By applying RA as a first step, we find that such relationships can be detected and ranked for their ratiometric stability.

We use a previously published large B-cell RNA-seq dataset from the 1000 Genomes Project Consortium [8] to characterize the ratiometric method. These data represent a rapidly growing class of RNA-seq data, in which many samples are biologically similar to each other. We reasoned that such datasets are incompletely served by the classical methods that focus on sample differences/perturbations to identify gene-sets that are co-regulated or otherwise function jointly. Among highly similar samples, we postulated that a hallmark of shared regulation and/or function would instead be constancy of relative expression levels (detected here pair-wise). We reasoned that this measure would be robust in a way that the usual measures would not be for both technical and biological reasons.

We revaluate that results from RA and find they are markedly different from correlation- and mutual information-based methods. RA captures relationships ignored by other measures and also assigns different rankings to many relationships detected by all methods. Expression relationships uniquely or preferentially detected by RA include important biological processes such as RNA processing. Compared with the traditional methods, RA also identifies a larger number of relationships that are annotated as KEGG pathways. Mere expression level similarity-by-chance did not explain this collection of functional relationships. We consider possible regulatory implications for ratio-stable genes and pathways and show that for RNA processing, which was the most RA-preferential pathway, many of the driving genes have been previously shown to be highly sensitive to quantitative variation or to be in a known ratiometric relationship. Finally, we perform a pilot test on single-cell RNA-seq data collected from a lymphoblastoid cell line, and show that RA performs more robustly than do conventional measures. We discuss how RA could make a unique contribution to emerging high throughput single-cell studies.

## Results and discussion

### A ratiometric approach for evaluating pairwise gene expression relationships

Considering a gene pair *A* and *B*, we define the ratiometric relationship as a function of the relative dispersion of the ratios *A*/*B* and *B*/*A*. This relative dispersion is measured by the coefficient of variation (*CV*), which is the standard deviation of the ratio divided by the mean of the ratio. The rationale for this choice is given in the Methods and Additional file 1: Supplemental Method section. To determine how well a ratiometric equation describes a gene pair’s expression pattern, we calculate the stringency of a fit to a ratiometric relationship as follows:



We use Δ_*CV*_ to explicitly model the variability in expression values for a given gene pair (A,B), which may affect traditional measures of co-expression differently than RA. This arises when variability is lowered because the range of values for A and B is restricted. Below we outline how Δ_*CV*_ relates to restriction in the variability of A and B and discuss why this can be important. We also note that value of Δ_*CV*_ is the same no matter what the order of the genes A and B is, as are the correlation coefficient and mutual information measures. Additional variability and its relationship to Δ_*CV*_ is discussed in Methods and Additional file 1.

Two genes are defined as following a ratiometric profile if Δ_*CV*_*<* 0.01. We selected this value based on prior analysis of the data structure by looking at the sensitivity of the results when changing the Δ_*CV*_ (Additional file 1: Figure S1), in addition to a simulation that examined the degree to which two independently expressed hypothetical genes would produce a false positive gene pair relationship (Additional file 1: Figures S2 and S3). Within the tested Δ_*CV*_ -range there was little or no change in RA gene ranking and the increased gene pair incorporation was mainly observed at the lower end of the stability range. The robust results in gene ranking were combined with the theoretical justification of a Δ_*CV*_ close to 0 (set stringently to reduce false negative gene pair associations). In any use, the Δ_*CV*_ would be adjusted to accommodate data with different characteristics based on the observed incorporation profile at different Δ_*CV*_ settings. Once gene pairs were selected by this criterion, further analyses were based on *CV*(*A*/*B*) and *CV*(*B*/*A*) that are below a specified value. It is convenient to specify these two ratios together as CV, as when Δ_*CV*_ is set close to 0 the two CVs are approximately the same. The smaller the CV, the stronger the ratiometric association (RA). In figures and tables, we abbreviate the ratiometric approach as RA (corresponding to the resulting gene pairs after first applying the stringency of fit, Δ_*CV*_ and then reporting the stability, CV, for those selected gene pairs, as described above), mutual information as MI (with mutual information statistic I), and the squared Pearson correlation coefficient as PE (with R^2^ as the statistic). We confine most analyses in the rest of the study to these three major models, though we note that the Spearman correlation coefficient was also tested for many of the analyses and behaved very similarly to Pearson R^2^.

### Variability and association

In addition to directly seeking ratio-based relationships the RA approach, as we have developed it, also focuses on variability. An underappreciated property of the standard statistical measure of association (correlation) is that it combines elements of both association and variability. This was noted by Bland and Altman [7], when they explored how restrictions in the range of gene expression leads to reduction in the correlation coefficient. They note that

“*Correlation coefficients are a property of the variables and also the population in which they are measured. If we look at a restricted population, we should not conclude that there is little or no relation between the variables because the correlation coefficient is small.*”

This observation is consistent with results from the statistical literature, as it is known that truncation (which changes the “population” referred to above), can alter the correlation coefficient of the bivariate normal [9]. While truncation (in their words, “restricted population”) is sufficient to cause the problem, it is not required, because simple narrowness of the range of expression can be enough to reduce the correlation coefficient. In principle, this condition would be expected in some biologically important parts of gene expression space, especially for high-quality data from samples that are quite similar to each other, where that similarity is driven by biological constraint and functional affiliation.

**Figure 1:**
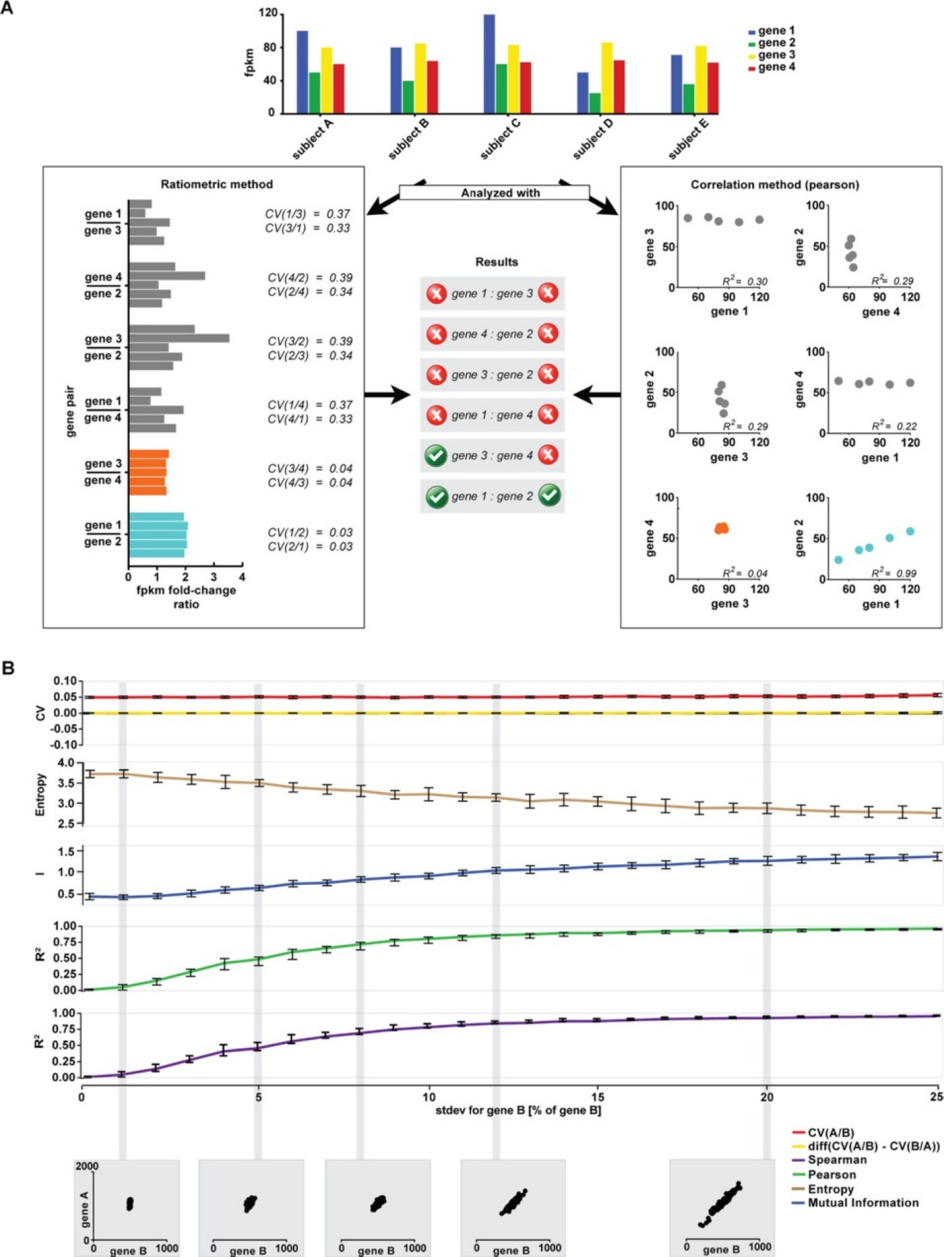
**Schematic illustration of the differences between the ratiometric analysis and correlation metrics. A)** For five samples (A-E), the expression levels of genes 1–4 are measured (top graph). The box on the right shows the analysis of expression relationships using a Pearson correlation. Only gene pair 1:2 is identified as a significant interaction, (R^2^ = 0.99). In contrast, the ratiometric method (box on the left) identifies both pairs 1:2 and 3:4 as significant. The PE method does not capture the second relationship (3:4) as the FPKM ranges of the two genes are too narrow for a regression line to be stable. On the other hand, the RA model assesses only the FPKM fold-change across samples, is much less sensitive to narrow FPKM ranges, and identifies both pairs. **B)** Shown is simulated expression data for two genes, A and B. The expression levels of *A* were generated from those of *B* as follows: *a*_*i*_ = 2*b*_*i*_ + *u*_*i*_. For each dataset, the expression range of B was varied by increasing *CV(B)* from 0 to 25% of the mean level of *B*(*μ*_*B*_, = 500). The expression level of *B* is thus normally distributed *B* ∼ *N*(500, % *B*). For each value of *CV(B)*, 10 datasets with 100 samples each were generated. The Pearson and Spearman R^2^, the entropy, mutual information, and the *CV(A/B)*, *CV(B/A)* and Δ_*CV*_ -values were calculated for each dataset, and the mean and standard error are shown. Note that the gene pair association does not change along the *x* axis and the expression of gene B can be used to predict the expression of gene A equally well in all runs. As the expression range for B narrows, the Pearson and Spearman R^2^-values decrease, along with the mutual information index. In contrast, the ratiometric CV is constant and the relationship between the expression levels of the two genes is always recovered.

We illustrate this with a “toy case” and a simulation (Figure 1A). A plausible pattern for 4 distinct genes across 5 samples, showing all possible relationships between them, is shown together with PE (R^2^) and RA (CV) analyses. PE evaluates the gene pairs based on the degree of covariance, while RA measures the stability of each ratio. In this hypothetical case, only gene pair (*1,2*) has a sufficiently high R^2^ to be considered correlated and these genes are also identified to be in a ratiometric expression relationship. However, an additional pair of genes (*3,4*) is also in a ratiometric relationship, but it is not detected using R^2^. This shows how, in principle, gene pairs can be undetected by the traditional PE method, but be detected by RA. The most notable difference between gene pair (*3,4*) and gene pair (*1,2*) in the above example is the narrow expression range of the former two genes compared to the latter two. We note that this is a situation one might especially anticipate among samples of similar type, such as similar individual cells, and that these underlying relationships might be biologically pertinent (see below).

Following this reasoning, we next examined the expression range sensitivity of PE, MI and RA by carrying out a simulation of expression data for two hypothetical genes, *A* and *B*. The expression levels of *A* are generated from *B* by the following equation for every sample *i*:


where *u*_*i*_ ∼ *N*(0, 50). For each run, we varied the expression range of *B* by increasing the standard deviation of *B* from 0 to 25% of the mean expression level of *B*. Setting E[*B*]=500, the expression level of *B* is thus normally distributed following *B* ∼ *N*(500, *s*^2^), where *s* is standard deviation ranging from 0 to 125 of the expression level. Each run was repeated 10 times with n=100, and for each iteration we calculated Pearson and Spearman R^2^ values, mutual information, *CV*(*B*/*A*), *CV*(*A*/*B*) and Δ_*CV*_ (Figure 1B). The Pearson and Spearman R^2^ and the mutual information index are very low at low *s*, and they only approach 1 (for PE and SP) and 2 (for MI) as *s* increases. In contrast, the *CV*(*A*/*B*) measures were constant throughout the whole range of *s*, and thus accurately described the underlying generative relationship of a constant association between the two genes. If we take “restriction” to mean, more generally, limited variation in one of the genes, these results confirm Bland and Altman’s concern and argue in favor of alternative approaches.

**Figure 2:**
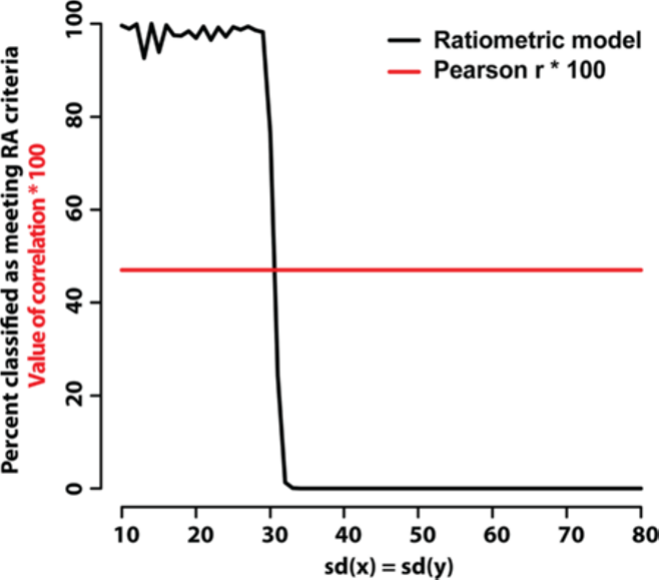
**Simulation for a bivariate normal distribution.** The fraction of observations (1000 runs of 1000 observations each for each value of the standard deviation) meeting the ratiometric definition (with a stability of 0.135) is shown as a function of the average correlation coefficient for each standard deviation. The constant correlation coefficient in this figure under the assumptions of equal changes in the standard deviation can be shown (using probability calculus) to be a property of the multivariate normal distribution.

Simulations also showed that the correlation coefficient can remain the same in situations where the ratiometric score varies, with the ratiometric measure once again being more sensitive in cases of low variability. Figure 2 shows results of a supporting simulation for a bivariate normal distribution with *E*[*A*]=*E*[*B*]=200 with a fixed Pearson correlation coefficient *r* = 0.47, with the standard deviations of both *A* and *B* allowed to vary from 10 to 80 while always being equal to each other. Agreement with the ratiometric selection criteria falls off rapidly once the standard deviation exceeds 30, while the correlation coefficient remains constant. Additional file 1: Figure S4 shows an example from the 1000 Genomes dataset of a pair of genes that has a low R^2^ but meets the RA criteria.

### Dataset collection and processing

To test the ratiometric method on a representative contemporary homogeneous sample group, we used a panel of RNA-seq datasets for lymphoblastoid cell lines derived from 462 human individuals from the 1000 Genomes Project [8]. This is a good test case because it consists of high-quality datasets over a large number of biologically similar samples. In this setting, we reasoned that biologically important gene pair relationships, driven by either direct or indirect co-regulation relationships, would display a low degree of expression fluctuation, since only one cell type and growth condition is represented in the study. However, we did not know what fraction of already known pathways and cellular structures would be identifiable by our criteria.

Raw sequencing reads were downloaded and quantified in a uniform way using eXpress [10] (see the Methods section for details). Genes with very low expression values were excluded so that only genes with FPKM values ≥1 in at least 95% of samples were included (Methods, Additional file 1: Figure S5). We note that in cells of this type, we have previously measured the number of transcripts per cell [11] and the threshold selected here is below the level that corresponds to one transcript per cell. Subsequent analysis was carried out on the resulting dataset.

### Relationships between RA, PE and MI

We examined the distributions of the Pearson correlation coefficient *r*, R^2^, Δ_*CV*_ and mutual information, I, and found them to have the same general shape, with the exception of the correlation coefficient (Additional file 1: Figure S6). This similarity in shape, however, does not extend to a similarity in the variation of these measures. As Table 1 reveals, I and R^2^ are very similar, with a correlation coefficient between the two >0.9. This is not unexpected as R^2^ can be interpreted as the fraction of variation explained, and mutual information as a likelihood ratio and hence goodness of fit. The simple Pearson r is moderately correlated with the I and R^2^ values. However, while Δ_*CV*_ is highly correlated with *CV*(*A*/*B*) and *CV*(*B*/*A*), none of these three measures is meaningfully correlated with the more usual measures of association. This suggests that the RA method is identifying a substantially different kind of relationships.

**Table 1:** **Correlation between measures**

	I	R ^2^	r	∆ _CV_	CV(A/B)	CV(B/A)
I	1	0.9125	0.451	-0.0833	-0.152	-0.139
R^2^	0.9125	1	0.631	-0.0587	-0.121	-0.117
r	0.4509	0.6308	1	-0.053	-0.144	-0.136
∆_CV_	-0.0833	-0.0587	-0.053	1	0.705	0.723
CV(A/B)	-0.1519	-0.1212	-0.144	0.7052	1	0.126
CV(B/A)	-0.1394	-0.1171	-0.136	0.7230	0.126	1

Finally, while reduction of variability is one way to meet our criteria for ratiometric behavior, it can also arise by following the ratiometric relationship *A*/*B* ≈ *c*. It is shown in the Additional file 1 (section “Analytical Analysis of the CV”) that a reduction of expected variation agrees with some intuitive definitions of ratiometric behavior (such as those found in [12]). An example of that type of reduction from a pair of genes from the 10000 Genomes project is shown in Additional file 1: Figure S7.

### The gene expression connectivity landscape

To investigate how the ratiometric approach evaluates gene pair relationships differently than the PE and MI methods, we ranked all gene pairs according to their association strength calculated by each method in the 1000 Genomes dataset. We then divided each ranking list into a series of 100 steps according to its stringency range (for PE: *R*^2^[1→0], for MI: *I*[2→0], and for RA: Δ_*CV*_[0→1]). Each step or set of relationships was drawn as an interaction graph, where the nodes are genes and the edges the gene pair relationships. We denote the graphs as *G*_*i*_ (where *i* is the stringency level index, with 1 corresponding to the most stringent set), the set of undirected edges in each graph (i.e. detected relationships between genes) as *E(G*_*i*_*)* and the number of such edges as |*E(G*_*i*_*)*|. We excluded all genes with a vertex degree *dg*(*v*=0) (no relationship detected) from each graph and denoted the size of the remaining vertices as |*V(G*_*i*_*)*| (see Additional file 1: Figure S8 for further details).

**Figure 3:**
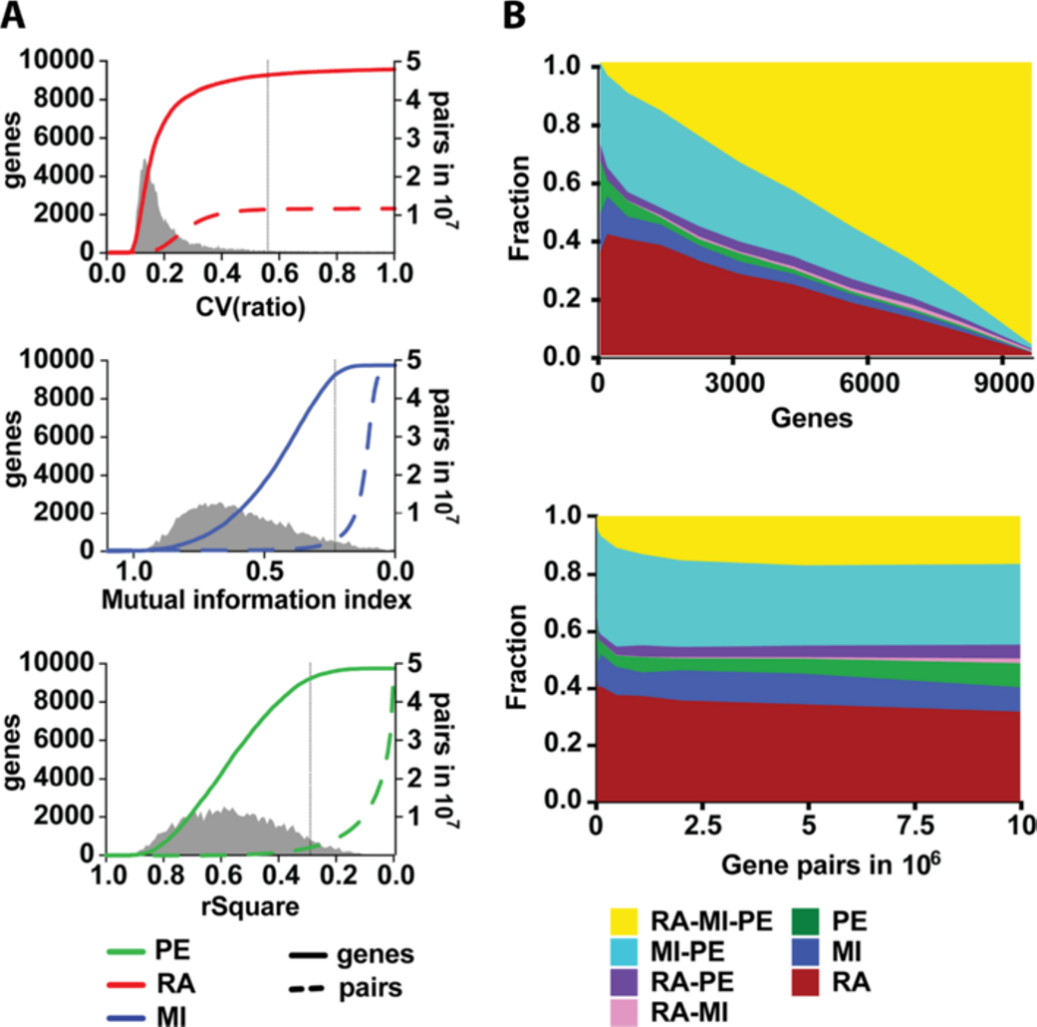
**Connectivity trends in gene graphs built by each model.** Analyzing the increase of connectivity coverage as the stringency level (coefficient cutoff) decreases. **A** and **B**, for every cutoff, the accepted portion of the graph is plotted as the percentage of all genes (solid line, left y-axis) and the number of gene pair connections (dashed line, right y-axis). All three graphs include >98% of the genes at the lowest plotted stringency level, though the dynamics of how the graphs grow are different, separating the RA method from PE and MI. Likewise in gene pair relationship inclusion, with the additional distinction that as the ratiometric model does only consider connections that exhibit a ratiometric profile it will have a smaller total number of possible connections (24% of total gene pairs possible). The dashed black vertical line marks the stringency level at which 95% of the genes are included, |*V*(*G*)| = 9244. B) The overlap of genes and gene pairs between the three methods at different vertex sizes, plotted against number of genes accepted (upper graph) or number of gene pairs accepted (lower graph). For each size of the graph, the following fractions of the total number of discoveries (genes or gene pairs) are given: RA, MI, PE, RA-MI, RA-PE, RA-MI-PE, MI-PE, where RA = ratiometric method, PE = squared Pearson correlation and MI = mutual information. PE and MI have a larger overlap compared to the RA method that differs compared to both former ones.

We first examined the number of individual genes participating in a RA, PE or MI relationship as a function of the ratio stability CV, R^2^ and I, respectively (Figure 3A, solid lines). Naturally, more genes are identified as the thresholds are relaxed (by increasing the CV and decreasing the R^2^ and I inclusion cut-off). The RA method displays a 2-stage inclusion profile, in which the majority of genes are included within a short interval at high stringency and inclusion then quickly levels off. The few remaining genes are spread out over a much larger range, as the stringency level decreases (gray filled curve in Figure 3A). PE differs, having an inclusion profile that is roughly bell-shaped, while MI behavior falls somewhere between the other two.

The distinction between the RA method and PE or MI is even more striking when the interactions among gene pairs are analyzed (Figure 3A, dashed lines). RA again displays rapid inclusion followed by a leveling off. In contrast, both the PE and MI methods exhibit exponential increases of interaction inclusion, but only at very low stringency levels that are much lower than the stringency at which the majority of genes had been identified as participating in at least one interaction. The implication is that when new genes are incorporated in RA clusters they form, to a greater extent, interactions with preexisting genes, and the number of new interactions identified increases rapidly relative to PE or MI clusters. When 95% of the genes (|*V*(*G*)| = 9244) are incorporated in a graph (Figure 3A, black vertical line), RA identifies ~5-fold and ~4-fold more gene interactions compared with the PE and MI approaches, respectively. Hence the bulk of the gene pair interactions are included in PE and MI clusters long after most genes are already added, while in RA the bulk of interactions are incorporated into the graphs together with genes.

The different RA inclusion profile would be of little biological relevance if the different methods produced identical gene and/or gene pair ordering. To investigate whether this is the case, we first generated Venn diagrams using same-sized graphs from the three methods for several different graph sizes, first using genes, |*V*(*G*_*i*_)_*RA*_| ≈ |*V*(*G*_*i*_)_*PE*_| ≈ |*V*(*G*_*i*_)_*MI*_|, and then using gene pairs, |*E*(*G*_*i*_)_*RA*_| ≈ |*E*(*G*_*i*_)_*PE*_| ≈ |*E*(*G*_*i*_)_*MI*_|, as size measures (Figure 3B, Additional file 1: Table S5 and Table S6). As expected, we observed a high degree of overlap between both gene sets and gene-pair sets produced by PE and MI. In contrast, RA found large numbers of genes and gene pair interactions per graph (and accordingly, per stringency level) that are unique relative to PE and MI. We therefore conclude that a separate set of gene expression relationships exist that are not identified as strong associations by traditional measures but are captured by RA.

### Invariant gene expression does not automatically lead to stable ratios

Some genes in our dataset are expected to, display constant ratios and so thus have a favorable RA-score, because they are invariantly expressed in the sample set, without reflecting any underlying biological relationship. To test if such constancy-by-chance relationships are prominent and could account for most of the ranking order produced by RA, each gene expression variation was plotted against its most stable ratio (Additional file 1: Figure S9). Invariant gene expression alone does not correlate strongly with high RA-ranking, *R*^2^=0.35, when including the top 7000 RA-ranked genes. Thus, RA rankings do not mainly reflect the general gene expression homogeneity in the dataset, but instead identify certain gene pair relationships based on more specific ratio stability.

### Characterization of gene pair relationships highly ranked only by PE and MI

To further assess the differences between the different methods, we carried out a GO term analysis of gene pairs that do not fit a ratiometric relationship (i.e. Δ_*CV*_*>*0.01) and are hence not RA-ranked at all, but are highly ranked by PE and MI. It revealed that they are enriched for genes involved in apoptosis, regulation of cell death and caspase activity (Additional file 1: Figure S10). Among the gene relationships highly ranked by PE and MI, ~40% display a 2-regime pattern in which a smaller subgroup is highly expressed and drives the high ranking, while the majority of the data points indicate a low expression with little correlation. No 2-regime gene pair relationships where discovered among those highly RA-ranked.

### GO category enrichment among ratiometrically related genes

A major question raised by these observations is the biological significance of ratiometric expression relationships, especially those that were not identified or were ranked as weak by standard PE and MI methods. We compiled the set of genes *V(G*_*i*_*)* in each RA, PE or MI graph *G*_*i*_ at varying vertex set sizes *i* = 1,…,5 (Additional file 1: Table S5 and Table S6) and used DAVID (http://david.abcc.ncifcrf.gov, [13, 14]) to assess GO category enrichment for each set, with the following modifications. Because a number of GO categories are functionally highly similar, we merged categories by grouping the genes in all branches in the GO tree hierarchy up to the level at which the resulting aggregated GO category contained more than 200 genes among the 9752 genes included in our lymphoblastoid RNA-seq data analysis. We consider an aggregated GO category to be enriched if the Bonferroni-corrected *p*-value ≤ 10^-4^. For easy reference the aggregated GO categories are further grouped into more general biological categories, e.g. “ribosome” and “cell cycle”.

**Figure 4:**
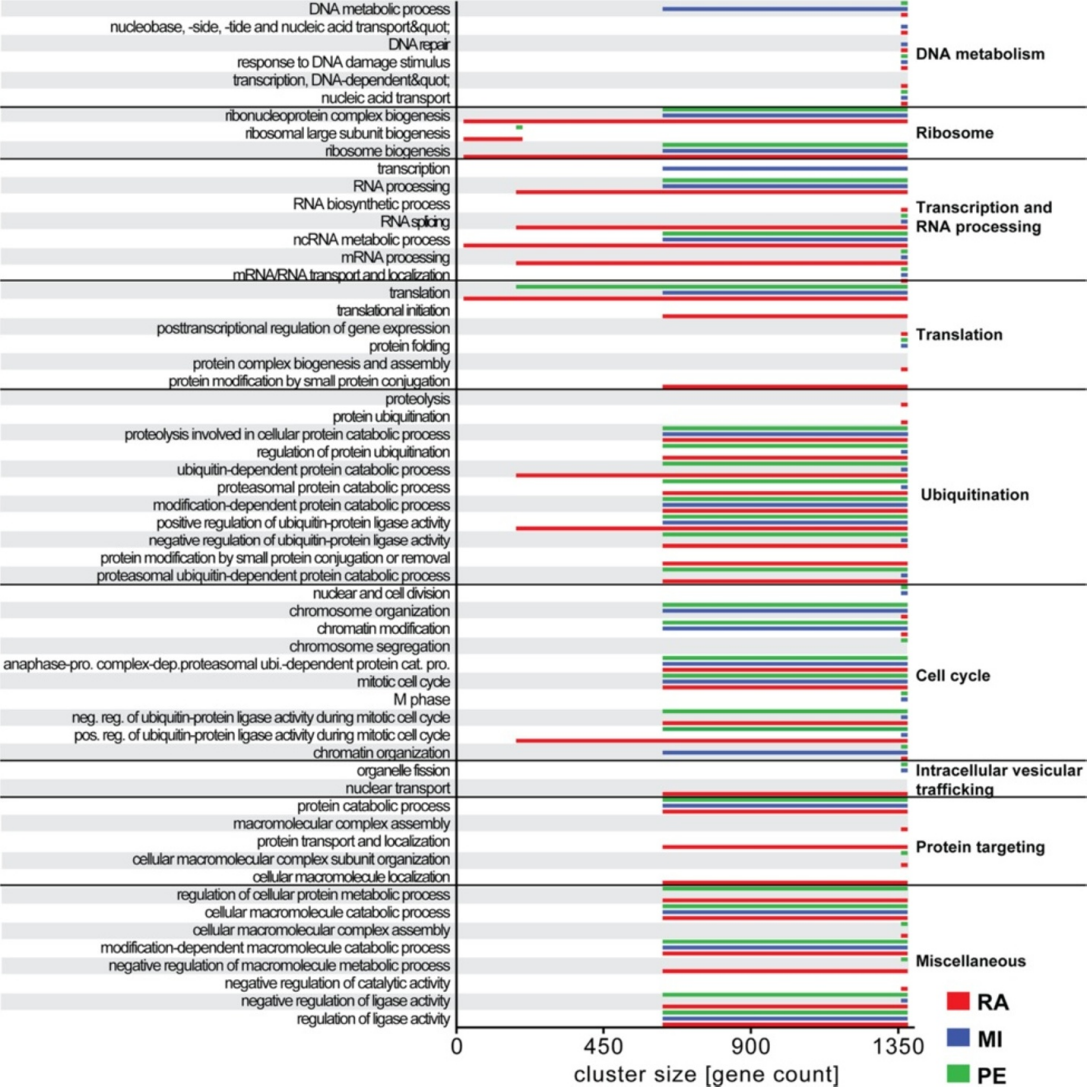
**GO category enrichment for the top vertex sets.** The V(G) sizes plotted for which, the aggregated GO categories (biological processes) have a GO enrichment with a Bonferroni-corrected *p ≤* 10^-4^. The aggregated GO categories are created by using the complete graph (|V(G)| = 9752) and grouping all GO categories with less than 200 detected genes together with its closest GO category upwards in the GO tree hierarchy having a detected gene set of ≥200.

Results of the GO analysis are summarized in Figure 4. The sets of genes in RA relationships were enriched for more GO categories than the sets identified using PE or MI. In addition, in cases where GO categories were enriched for all three, ratiometrically-constructed graphs generally exhibited enrichment at higher stringency levels (i.e. smaller |*V(G*_*i*_*)*| and lower *i*). RA displayed higher enrichment for ribosomal genes, genes involved in transcription and RNA processing, translation, and ubiquitination. By contrast, PE and MI sets were more highly enriched for only one function, the aggregated cell cycle-related terms.

### KEGG pathway analysis

**Figure 5:**
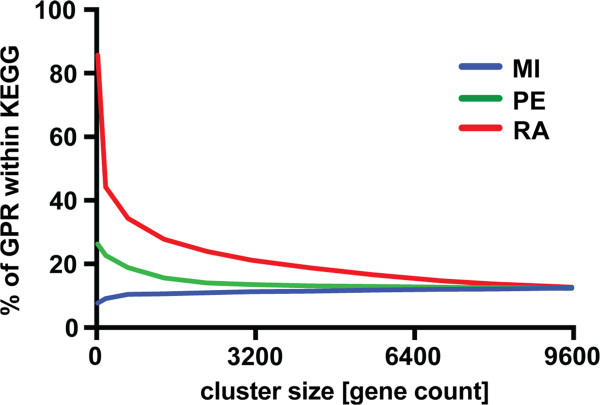
**Fraction of gene pair relationships between annotated and non-annotated genes in KEGG detected by each method.** The percentage of gene pair relationships (GPR) between two annotated genes in KEGG of the total number of relationships reported for each graph size (given in number of genes included). The RA method detects to a higher degree than PE and MI gene pair relationships that are within the KEGG annotated database.

We further explored the biological relevance of the ratiometric relationships by using the pathway annotations in the Kyoto Encyclopedia of Genes and Genomes database (KEGG, (http://www.kegg.jp, [15]). We calculated the percentage of edges in each graph that are between genes both of which are annotated in a KEGG pathway, at similar graph vertex set sizes |*V(G)*| for RA, PE and MI. This analysis revealed first, that ratiometric relationships are more enriched in KEGG interactions, and second, that the strongest enrichment differences between the methods are at high-inclusion stringency levels (i.e. small |*V(G)*|) (Figure 5). The ratiometric analysis discovered more validated biologically relevant interactions than PE and MI among the top-ranked gene pairs.

**Figure 6:**
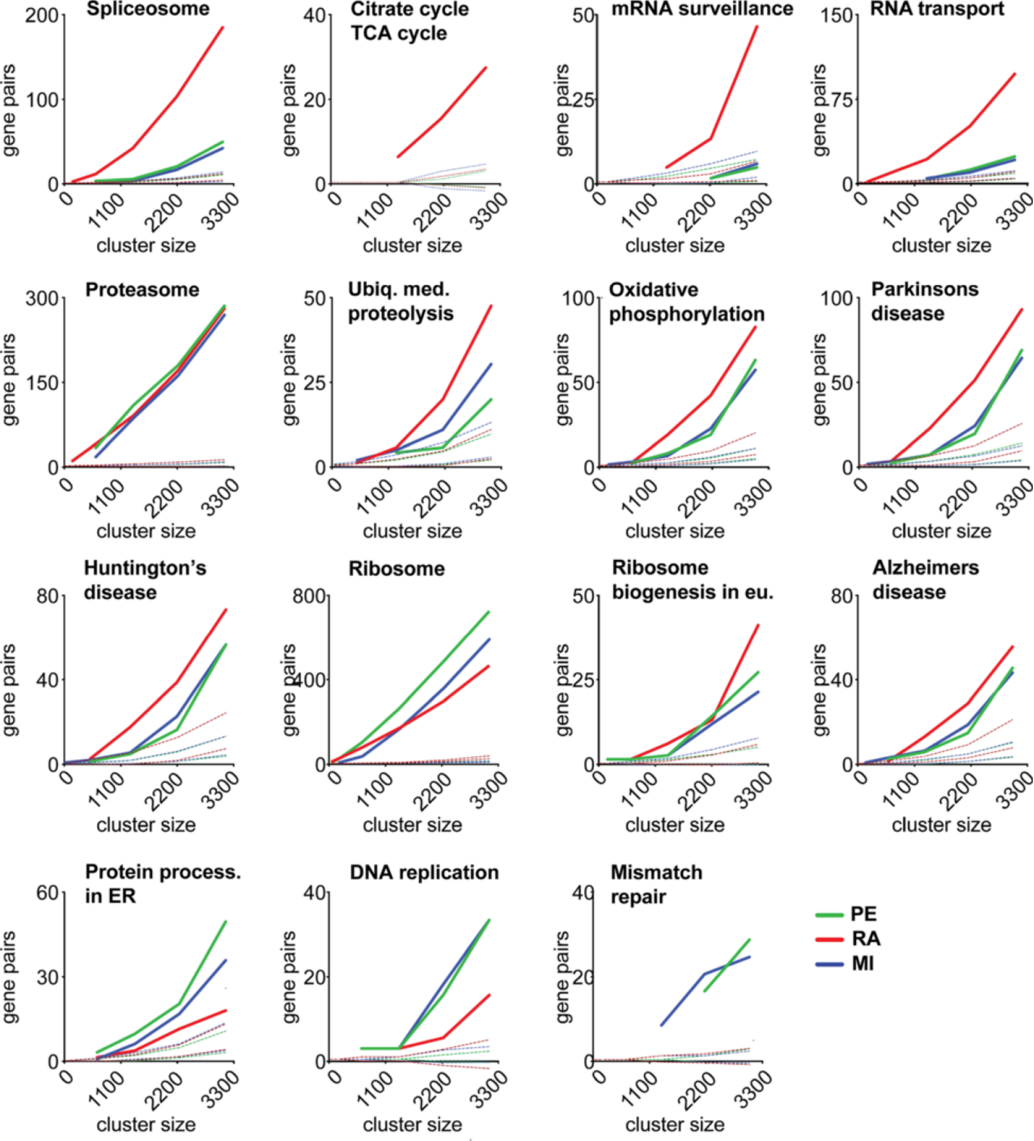
**KEGG pathway enrichment analysis.** The number of gene pair relationships within the same pathway plotted against an increasing graph size, given in gene number. The RA method, red, MI, blue, PE, green. Only pathways with gene % ≥25 for at least one method at graph size ~3000 genes are included in the figure. Pathways detected early in the graphs are: ribosome, proteasome, spliceosome, RNA transport. Pathways picked up by the RA method more strongly than PE and MI are both basic cell function pathways as well as disease related ones, such as: spliceosome, RNA transport, mRNA surveillance, and citrate cycle TCA cycle. Slightly stronger: proteasome, ubiquitin mediated proteolysis, Parkinson’s disease, oxidative phosphorylation, and Huntington’s disease. Pathways picked up equally well by all three methods are ribosome, ribosome biogenesis in eukaryotes, and Alzheimer’s disease. Where PE and MI methods do better than the RA model is in pathways protein processing in ER, mismatch repair, and DNA replication. Dashed lines represent the perturbed runs simulating the actual pathway.

Next, we analyzed the recovery of individual KEGG pathways by the three methods, using a similar framework. We calculated the number of edges within a KEGG pathway identified as a function of |*V(G)*|. Only KEGG pathways for which at least 25% of the genes were part of at least one of the three graphs at |*V*(*G*)|=3100 were considered. The ratiometric analysis identified more interactions than PE or MI for most KEGG pathways (Figure 6).

**Figure 7:**
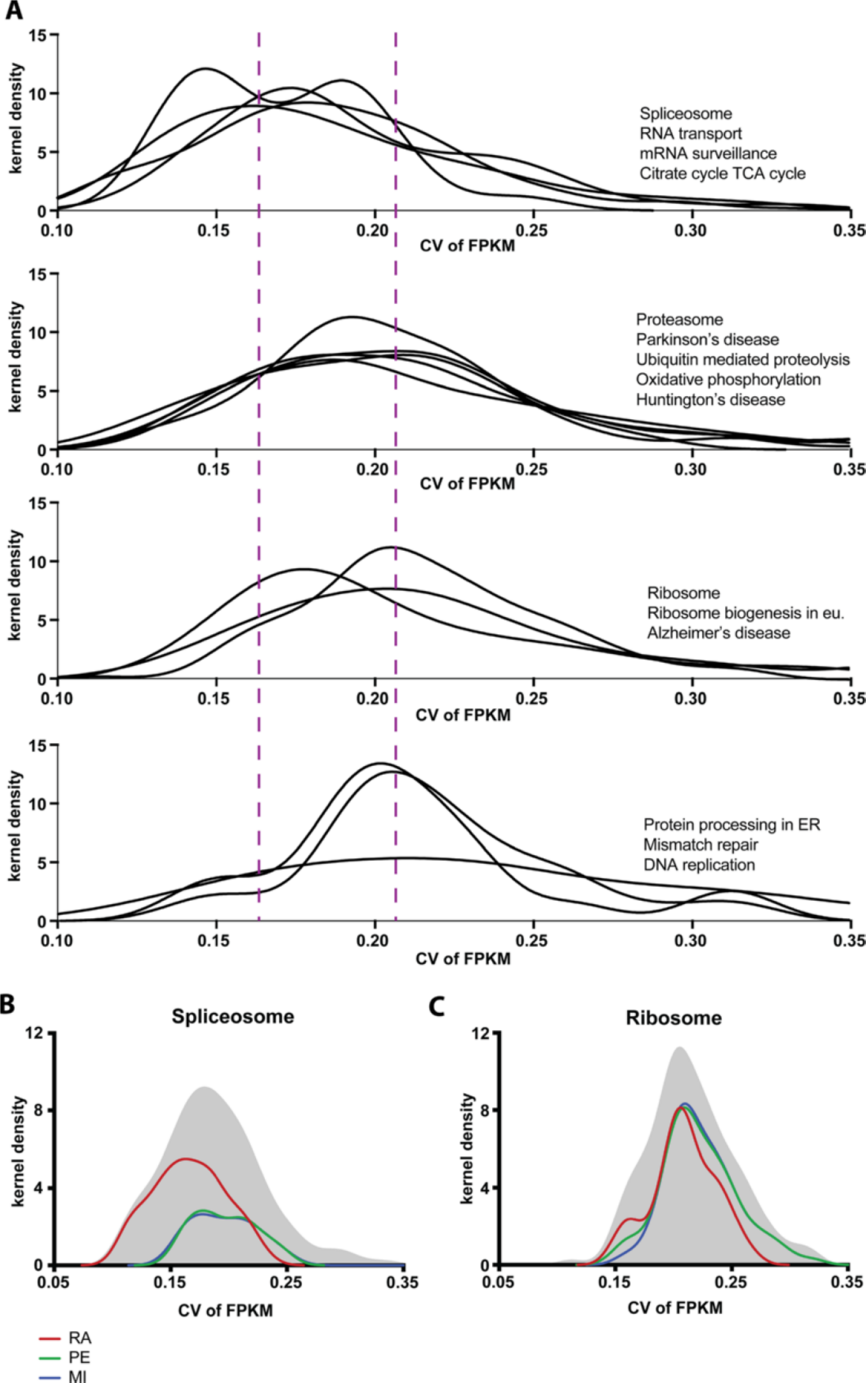
**Distribution of FPKM CVs per KEGG pathway. A)** The kernel density, per pathway, of FPKM CVs for the pathway’s genes found in our data set. The Top graph show pathways detected much more strongly by the RA method than by the PE and MI. The second graph show pathways picked up slightly more strongly by RA. The third graph show pathways detected equally well by all three methods. The bottom graph contains pathways better picked up by PE and MI compared to the RA method. The better the RA method detects a pathway compared to the other two, the further left its curve sits. The dotted purple lines indicate how the distributions shift towards the left as one goes up the graph series. **B** and **C**, Two pathways, one from the top graph, and one from the third graph, showing the densities for the subgroup of genes that each method detects at V(G)_5_ = 2220. The pathway CV(FPKM) density (gray), RA (red), MI (blue), PE (green).

We then investigated the possible reasons for the observed differences in pathway recovery by the three methods. As suggested above, a potential strength of the RA approach compared to PE and MI is the ability to identify relationships between genes having narrow expression ranges. We therefore examined the gene expression ranges for genes involved in each pathway (Additional file 1: Figure S11). We divided pathways into four groups: 1) pathways that the RA approach recovers better than PE or MI by a wide margin; 2) pathways recovered similarly by all methods, but slightly better by the RA analysis; 3) pathways for which the three methods identify an approximately equal number of genes; and 4) pathways better recovered by PE and MI, Figure 7A. The *CV*(*FPKM*) values are lowest, on average, for pathways in the first group, and highest for pathways that are better recovered by PE and MI, in agreement with prior theoretical considerations and simulation results (Figure 1B). In the Additional file 1, in the section “Statistical test of CV(FPKM) groupings” we show that group 1 has a significantly smaller (at less than the .001 level) average CV(FPKM) than either group 2, 3 or 4 and that group 4 is significantly larger (at less than the .005 level) than either group 1, 2 or 3 (groups 2 and 3 are indistinguishable statistically from each other). The results show that the pathways unique to the RA approach correspond to pathways with the lowest intrinsic variability across samples.

We further investigated the relationship between the expression ranges of genes within a given pathway and the interaction recovery rate. We began by comparing two major KEGG pathways: 1)“Ribosome”, which belongs to the group recovered equally well by all three methods, and 2) “Spliceosome”, which is recovered best by ratiometric analysis, Figure 7B. For the “Ribosome pathway”, all three *CV*(*FPKM*) distributions of the captured genes overlap. In contrast, the few genes in the “Spliceosome” recovered by PE and MI had broader expression range distributions compared with the spliceosome genes detected by RA.To ascertain that the KEGG enrichments reported by RA were not generated mainly by random pairing of genes that exhibit invariant expression across the samples, we performed a perturbation analysis. We generated randomly permuted models of each KEGG pathway, mimicking the number of genes in the pathway and its their RNA expression levels. We then calculated the detection rates by RA, PE and MI (Figure 6, dashed lines). We never observed a RA detection rate as high as that for known biological pathways, although some relationships are identified that are without an underlying KEGG pathway relationship. These results support the conclusion that RA relationships detected in our study often reflect known biological pathways, including some missed by the major existing approaches.

### IntPath gene pair relationship enrichment

**Figure 8:**
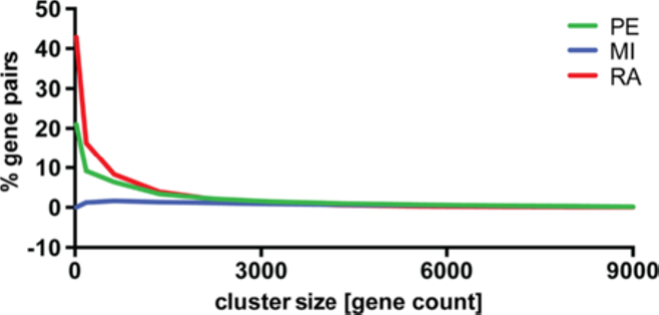
**Enrichment of IntPath-gene pair relationships at various stringency levels for the three methods.** The enrichment is given in percentage of gene pairs at a given stringency level that are annotated in the sapiensIntPathGenePairs database. RA shows a higher enrichment than Pearson correlation and mutual information.

To further verify the biological relevance of the gene pair relationships detected by RA, we used gene pair relationships annotated in the IntPath database (sapiensIntPathGenePairs, downloaded 16^th^ of May 2014) [16]. Again, RA produced a higher enrichment in IntPath-gene pair relationships among top-ranked genes compared to PE and MI (Figure 8). There was an overlap of pathways detected using KEGG and IntPath, with the two best identified being the ribosomal genes and the spliceosomal genes, with the latter annotated in KEGG as “Spliceosome” and in IntPath as “mRNA processing”.

### Application of RA to single-cell RNA-seq data

**Figure 9:**
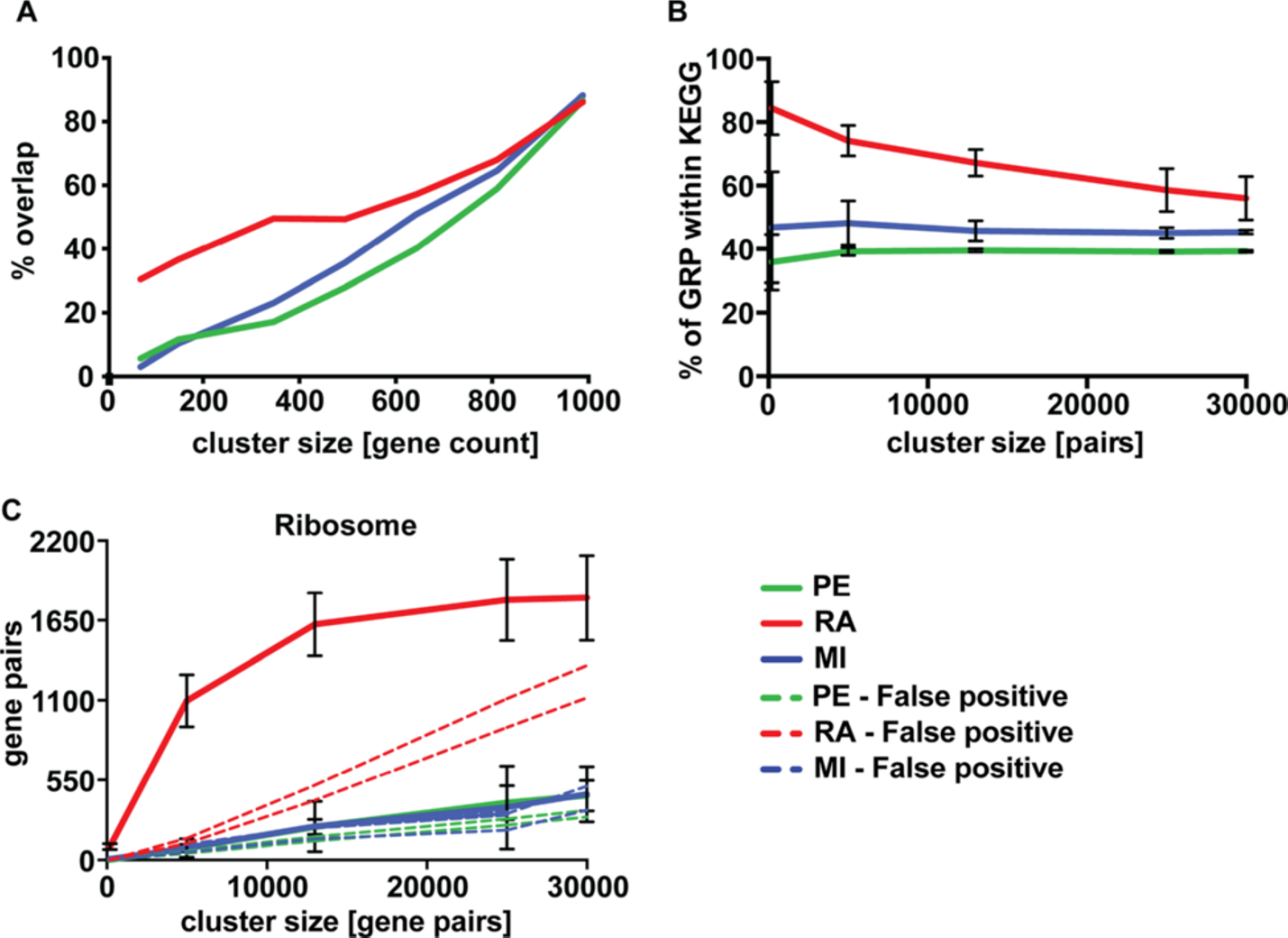
**Single-cell RNA-seq analysis.** Comparing the results from two separate batches of 10 single-cell samples, including 782 genes expressed in both batches. **A)** Ranking stability between the two batches for RA, MI and PE. **B)** KEGG enrichment per graph size. Error bars describe the variability between batches. **C)** The only KEGG pathway sufficiently represented in this data set is the Ribosome. The number of gene pair relationships reported within the ribosome pathway per graph sizes (solid lines) and the false discovery interval (dashed lines). Colors as RA (Ratiometric method), red, PE (Pearson correlation), green, MI (Mutual information), blue.

Single-cell RNA-seq is an emerging area in which many similar samples (soon hundreds or even thousands of similar cells per study) will be the norm. We therefore performed a pilot study to determine RA’s utility for analyzing this type of data compared to PE and MI. We applied it to two single-cell data sets of 10 RNA-seq libraries each from one of the 1000 genomes lymphoblastoid lines [11] as a pilot study (∆_CV_ = 0.025). We compiled a list of 782 genes expressed in at least 18 of the 20 cells (allowing one non-expression per data set) and then analyzed the two sets of single cells separately in order to measure the degree of consistency between the results for each method. First we analyzed ranking congruency of each method between the two sets, recognizing that these data are first-generation and that they display significant levels of known technical stochasticity [11]. RA displayed a higher degree of ranking consistency, *>40%*, between data sets (Figure 9A) than PE and MI. The RA associations were also more highly enriched by KEGG annotated genes compared to PE and MI (Figure 9B). When examining the individual pathways, the only one sufficiently represented among the 782 genes used for evaluation was the ribosome. Compared to bulk-RNA cell line data, where the “Ribosome” was easily detected by all three methods, only RA detected it in the single-cell data (Figure 9C). Thus RA dealt best with the considerable technical weaknesses of first-generation single-cell data [11]. At present, RA proved to be the most robust method by the criterion of ranking consistency and by its ability to identify relationships known from the larger bulk RNA data.

## Conclusions

The ratiometric approach developed here was able to extract gene expression relationships from a large set of RNA-seq samples from a single homogeneous cell type. These gene pair relationships were mainly not identified or highly ranked by the more traditional methods. We did this by using the relative dispersion of the expression ratios between pairs of genes to detect gene pair relationships. The ranking produced by RA is distinct from R^2^ (PE) and Mutual information (I), which produce results similar to each other. A striking result is that when comparing the top-ranked gene pair relationships across a large set of lymphoblastoid samples, the ones identified by RA were more highly enriched for biologically validated interactions than PE and MI.

RA has a straightforward biological interpretation, as it evaluates all possible gene pair interactions based on the stability of the ratio between their expression levels, regardless of the degree of dispersion of absolute expression values among the samples. In this sense RA is a simple and general interpretation of a gene (or transcript) pair association. PE and MI both conform to a notion of coordinated gene expression changes across samples, but they do it in a way that is insensitive to genes with narrow expression ranges. This means that certain ratiometrically constrained relationships would be missed. To the extent that many studies have explicitly or implicitly “cared” only about genes and pathways whose absolute levels differ substantially within a set of samples, PE and MI have clearly been very effective. They are at their best when variation between samples in the analysis is large. The resulting expression relationships, along with their gene network structures and regulatory mechanisms, have received much attention. RA’s particular strengths, on the other hand, are in detecting relationships between genes having by low dispersion, across a sample set. They have not received similarly intense attention.

Across the 1000 Genome B-cell RNA-seq data, GO category, KEGG pathway, and IntPath analyses showed enhanced recovery of known biological gene interactions by RA compared to PE and MI. This was most obvious for biological processes and complexes containing genes with low expression dispersion. By specifically comparing genes encoding components of the “Ribosome” and the “Spliceosome”, we explored the relationship between decreased detection power by PE or MI and narrower FPKM ranges. Genes in the ribosome pathway showed a wider range of relative expression values. The ribosomal pathway was identified equally well by RA, PE and MI, but the specific gene pairs responsible for RA enrichment differed from those responsible for PE or MI enrichment. In contrast, the genes in the spliceosome pathway displayed narrower expression ranges, leading this pathway to be scored as largely “non-associated” by PE and MI. RA analysis showed that a substantial set of spliceosomal genes displays an especially constrained relative expression pattern across these samples. This is consistent with previous literature suggesting that relative constancy may be important for defining a cellular state and that deviation from it may be important in some cancers [17–19]. Of particular relevance to our study is evidence that the relative levels of some RNA splicing factors determine alternative splicing patterns [20, 21]. RA assigned top-rankings to components of the RNA processing machinery that include HNRNPK [22], SRSFs1, 3, and 7 [17], TRA2β [23] and RBMX [24]. Although the relevant gene pairs have yet to be systematically perturbed, individual studies in various cell systems suggest quantitative sensitivity. For example TRA2β and RBMX are reported to regulate alternative splice site choices according to their ratio [24], and their protein products interact with each other [25] and with SRSF1. The SR and HnRNP classes are widely understood to compete to set isoform choice [26], and a recent example showed that the SRSF1 ratio relative to HNRNPA1 governs specific splice site choice in vivo [27]. Other high ratiometric rankings might be due to functional relationships other than RNA splicing among some of these genes. Thus RBMX can also function in homologous recombination initiated by DNA-damage [27], and it pairs strongly by RA rank with HNRNPK, which functions in the p53/TP53 DNA damage response [28].

Combining RA with either PE or MI revealed expression subdivisions within the sample group. In this specific case, the division appeared to correlate with apoptosis and regulation of cell death, suggesting that a small set of the cell line cultures were probably stressed. More broadly, we imagine that for the majority of datasets the optimal analysis will first use one of the traditional metrics as well as RA to distinguish potentially existing subgroups within the dataset. Based on those results, one might then move to RA for the entire set if it proves highly homogeneous, or instead parse it into informative sample subsets for RA: these sample subsets can then be reanalyzed by RA as individual groups (for example, specific cell types that have come from a diverse admixture sample of unknown starting composition). This stratified approach would focus RA analysis on internal relationships that meet useful ratiometric and associated variability criteria. This Such an approach would allow RA to identify gene pair relationships present across different cell conditions that have gone undetected by the major methods (three examples are given in Additional file 1: Figure S12).

In summary, RA allowed us to analyze homogeneous datasets and successfully identify in them expression relationships that are strongly invariant between individual samples. The relationships recovered were strongly enriched for biologically meaningful pathways and gene groups, based on GO, KEGG, and IntPath enrichment. We found that some of these relationships are ignored or low-ranked by conventional correlation and mutual information-based methods. This raises major questions for the future about the mechanistic basis of narrow-variance control, and about its possible implications for gene network structure and circuit function. Single-cell studies, now in their infancy, present additional questions about dynamic variation and stochasticity that were previously obscured in population data and for which RA may be especially well suited.

## Methods

### Data processing, gene expression quantification and gene set filtering

We downloaded publicly available lymphoblastoid cell line RNA-seq datasets generated by the 1000 Genomes Project Consortium (http://www.ebi.ac.uk/arrayexpress/experiments/E-GEUV-1/samples.htm, [8]). We aligned reads against the refSeq [29] transcriptome (generated using custom-written scripts for the hg19 version of the human genome) using Bowtie 0.12.7 [30]. The two ends of paired end datasets were aligned jointly with the following settings: \verb|’-aS -X 800 e -200 --offrate 1 --best –strata’| settings. The alignments were quantified using eXpress, version 1.3.0 [10]. In cases where multiple isoforms are annotated for the same gene, the expression values for all isoforms were summed to derive the final gene-level quantification estimate.

We filtered the list of genes to be included in the analysis by expression values in order to eliminate potential artifacts due to the inclusion of genes with very low expression levels. We included all genes having an FPKM value ≥1 for at least 95% of the samples, a total of 9752 genes. Setting the FPKM cut-off differently (0.1 and 5 respectively) altered the set of gene pairs reported to a lesser extent for the RA method compared to PE and MI, see Additional file 1: Figure S5. For example, the stringency level at which the most stringent FPKM cut-off ≥5 FPKM includes 225,093 gene pair relationships applying PE, includes 410,168 with the most lenient FPKM cut-off of 0.1, an increase of 82%. For MI the same calculations give an increase of 82%, while for the RA method the increase is only 12%. The RA method is therefore less sensitive to inclusion or exclusion of genes with low FPKM values.

### Ratiometric behavior from a statistical perspective

We refer to the model we present here as “ratiometric” because it is based on the ratios *A*/*B* and *B*/*A*. As described above, this method handles well low variation in the range of input gene expression values. There is another type of reduced variation that is picked up by our method, which is reduction of variation relative to what would otherwise be expected. As described above, if we have two genes A and B, if these genes are ratiometrically related by *A*/*B*=*c*, any paired set of observations from one sample (call them *a* and *b*) would seem to be related by the Equation *a*/*b*=*c*, where *c* is a constant. Similarly, under this assumption, *b*/*a*=*d*=1/*c*.

The analysis of the distribution of ratios is widespread in the statistical literature ( [31, 32] and their use is also not new in the biological literature [33]). It is, on the other hand, difficult to find studies in the statistical literature that attempt to test whether the relationship of two variables is in fact ratiometric, as even survey articles of models for the relationships of one variable to another tend not to include specifications of this form (see [34] and [35], for example).

One attempt to model the ratiometric behavior of two variables and offer statistical tests for the presence of such behavior was presented by Schnute [12]. The Schnute model assumes that every pair of observations of two variables is generated from a bivariate normal distribution where the mean of the first variable is equal to a constant times the mean of the second variable. This means that *E*[*A*] = *cE*[*B*], or rewriting, *E*[*A*]/*E*[*B*]= *c*, which is similar to our ratiometric specification above. In fact, in the Additional file 1: Supplemental Methods section we show that E[A]/E[B] = E[A/B] implies that the limit of Δ_*cv*_ = 0 under some conditions, so that there is a direct connection between the Schnute model and our ratiometric model.

The Schnute model is a statistical model; that is, it has an error structure, and in the Schnute model the error structure comes from an assumption of the distribution of any particular realization of the two variables being bivariate normal (the overall distribution of the A and B may not be bivariately normal, since the means may vary). The Schnute model is not identified, unless one or both of the gene pairs is non-normal, in which case methods for finding a *c* which allows the linear combination *A*-*cB* to be distributed normally allow identification of the c (a similar identification condition relying on non-normality exists for the errors in variables problem, a fact that Schnute notes).

Most gene pairs in the 1000 Genome dataset, though, are in fact normal or transformable to normal, so that in most cases the Schnute model does not apply. As noted above, a statistical model has an error structure, and *A*/*B*=*c*, which is our working definition of a ratiometric distribution, does not. An obvious step to take is to minimize the sum of squares of the observations, that is, , where the subscript indicates individual observations. In addition, if *A* and *B* are assumed to be in a ratiometric relationship, there is no particular reason to prefer one ratio (*A*/*B*) to the other (*B*/*A*), and so we would like to also minimize ∑(*b*_*i*_/*a*_*i*_ - *d*)^2^.

To compare these two minimizations, note that both of these expressions are simply the variance of (*A*/*B*) and (*B*/*A*). To make these variances comparable to each other, we note (described in further detail in the Additional file 1: Supplemental Method section) that there is reason to believe that these standard deviations, divided by their respective means (*E*[*A*/B] and *E*[*B*/*A*]), should be close to one another if the ratiometric relationship holds. The smaller the variation of the ratio with respect to the mean, the closer it is in a relative sense to the relationship *A*/*B*=*c*. We do not adopt a formal error structure on this problem. Rather, we use the criteria of varying the stability (the magnitudes of *CV*=(*A*/*B*) and *CV*=(*B*/*A*)) and making comparisons with other measures (such as the PE and RA). The criteria used for the actual gene pair selections in RA are thus based both on the theoretical behavior of the CV’s and on our empirical experience from analyzing the datasets. In Additional file 1: Figure S7 we show an example from the 1000 Genomes Project data which suggests how the closeness of a gene pair to a ratiometric relationship might be amenable to analysis through a statistical model, although we do not develop this any further in this paper.

We note that the coefficient of variation of the ratio has been used before as a measure of variability of gene expression, but only to test the difference of RNA values for the same gene between samples [33] and under the assumption of log-normality of the two genes. That differs from the use in this paper, since we use the various ratiometric measures to classify gene pairs, creating an alternate measure of co-expression.

### GO category enrichment analysis

We used DAVID (http://david.abcc.ncifcrf.gov, [13, 14]) to assess GO enrichment, with the modification that the nodes of the GO tree were merged until the resulting aggregated GO categories contained at least 200 genes that were also part of the set of 9752 genes included in analysis. We used a Bonferroni-corrected *p*-value of 10^-4^ as a significance threshold for enrichment.

### KEGG pathway analysis

We downloaded the annotated gene lists for each KEGG pathway from http://www.kegg.jp[15]. We calculated the enrichment of KEGG pathway interactions in Figure 5 by counting the number of graph edges in each graph *G*_*i*_ for which both genes were annotated in a pathway in the KEGG database. The same approach was used to calculate the percentage of genes recovered at increasing graph size |*V(G*_*i*_*)*| in Figure 6 for each KEGG pathway. Only KEGG pathways with a gene percentage result of 25% or more at |*V(G)*| = 3100 are shown.

### IntPath analysis

We downloaded the IntPath database *sapiensIntPathGenePairs* (16^th^ of May 2014) [16]. We calculated the enrichment of IntPath-relationships in Figure 8 by taking the percentage of graph edges in each graph *G*_*i*_ that are annotated in *sapiensIntPathGenePairs*.

## Electronic supplementary material

Additional file 1: **Supplementary Information.** (PDF 2 MB)
