# Supplementary material for: A ratiometric-based measure of gene co-expression
Source: BMC Bioinformatics. 2014 Nov 20;15(1):331. doi: 10.1186/1471-2105-15-331 (PMC4289233; doi:10.1186/1471-2105-15-331)
Supplement: Supplementary file 1 — Additional file 1: Supplementary Information. (PDF 2 MB) [file 12859_2014_6778_MOESM1_ESM.pdf]

A ratiometric-based measure of gene co-expression
Supplementary Information

Anna C.T. Abelin[1], Georgi K. Marinov[1], Brian A. Williams[1], Ken McCue[1,‡], and Barbara J. Wold[1,‡]

[1] *Division of Biology and Biological Engineering, California Institute of Technology, Pasadena, CA 91125, USA*

Supplementary Methods

Analytical Analysis of the CV

We explore several models to show the behavior of the CV's of the ratios. Essentially, under certain assumptions and conditions, it can be shown the that CV(x/y) and CV(y/x) approach each other. In particular, this equivalency can be independent of the variation of x and y, as is shown in some simulations at the end of this supplement.

Given two genes g and h, let $\tilde{x}$ be the expression level of gene g and $\tilde{y}$ be the expression level of gene h. Then g and h are described as being in a ratio relationship if

$$\frac{\tilde{y}}{\tilde{x}} = c + u, \qquad (1)$$

where c is the ratio of the expression levels of the two genes and u is an error term which is uncorrelated with any other variable. Then if x and y are the observed expression levels for g and h, one has that

$$x = \tilde{x} + u_x \text{ and } y = \tilde{y} + u_y. \qquad (2)$$

Since the sampling scheme in any single RNA-seq run can be presumed to be multinomial, and the accumulation of reads at any single gene is miniscule with respect to the total number of reads, a normal approximation to the distribution of this error term can be made under a Poisson sampling scheme, so that $\text{Var}(u_x) \approx x$ and $\text{Var}(u_y) \approx y$, with both of course having zero expectation.

Rewriting (1) as

$$\tilde{y} = \tilde{x}c + \tilde{x}u,$$

one can use the equalities in (2) to obtain

$$\begin{aligned} \tilde{y} &= \tilde{x}c + \tilde{x}u, \\ y + u_y &= (x - u_x)c + (x - u_x)u, \\ \frac{y}{x} &= c + u + \frac{1}{x}\left(u_y - cu_x - u_x u\right). \end{aligned}$$

Only the variance of the u (call it σ_u^2) is unknown so it is relatively simple to estimate this. Note that the above gives

$$\begin{aligned} \tilde{y} &= \tilde{x}c + \tilde{x}u, \\ 1 &= c\frac{\tilde{x}}{\tilde{y}} + u\frac{\tilde{x}}{\tilde{y}} \\ \frac{\tilde{x}}{\tilde{y}} &= \frac{1}{c} - \frac{u}{c}\frac{\tilde{x}}{\tilde{y}}, \\ \frac{\tilde{x}}{\tilde{y}} &\approx \frac{1}{c} - \frac{u}{c^2}, \\ \frac{\tilde{x}}{\tilde{y}} &= d + v \end{aligned}$$

the next to last step coming since $\tilde{x}/\tilde{y} \approx 1/c$ and setting $d = 1/c$ and $v = u/c^2$.[1] Estimation can then proceed as outlined above for $\tilde{y}/\tilde{x}$.

So $\tilde{x}/\tilde{y}$ will have a coefficient for the ratio which is the reciprocal of the $\tilde{y}/\tilde{x}$ expression and has the same error term scaled by $1/c^2$. Thus if estimation is performed when this is the true model, one would expect that

$$\begin{aligned} \frac{\hat{\sigma}_v}{\hat{d}} &\approx \frac{\sigma_u}{c^2}\frac{1}{1/c} \\ &= \frac{\sigma_u}{c}. \end{aligned}$$

The above ratios are actually the coefficient of variations of the ratios $\tilde{y}/\tilde{x}$ and $\tilde{x}/\tilde{y}$. Thus the two coefficients should be approximately the same when the ratio regime describes the relationship between the data.

We now use the Delta method (based on Slutsky's theorem (Bickel & Doksum 1997)) to demonstrate asymptotic equivalence of estimators of the CV's under a ratiometric regime. This demonstration depends on $\text{E}[y/x]$ being close to $\text{E}[y]/\text{E}[x]$, a condition that would seem to be satisfied by most ratiometric specifications, since $y/x = c$ implies $\text{E}[x/y] = c$ but also that $y = cx$ so that $\text{E}[y] = c\text{E}[x]$ or $\text{E}[y]/\text{E}[x] = c = \text{E}[y/x]$.

Define μ_x and μ_y ($(\mu_x, \mu_y) = \mu$) as the means of x and y, so that μ is asymptotically normal and has covariance matrix Σ. Then by the usual corollary to Slutsky's theorem, any function $f(x, y)$ is also asymptotically normal with mean $f(\mu_x, \mu_y)$ and variance $\nabla f(\mu)^t \Sigma \nabla f(\mu)$. Setting $r(a, b) = a/b$ and $s(b, a) = b/a$, the asymptotic means of these two functions are $r(\mu_x, \mu_y) = \mu_x/\mu_y$ and $s(\mu_x, \mu_y) = \mu_y/\mu_x$. Variances are given by

$$\begin{aligned} \text{Var}\ (r(x,y)) &= \left[\begin{array}{cc} \frac{1}{\mu_y} & -\frac{\mu_x}{\mu_y^2} \end{array}\right] \Sigma \left[\begin{array}{cc} \frac{1}{\mu_y} & -\frac{\mu_x}{\mu_y^2} \end{array}\right]^t \\ &= \frac{1}{\mu_y^2}\left[\begin{array}{cc} 1 & -\frac{\mu_x}{\mu_y} \end{array}\right] \Sigma \left[\begin{array}{cc} \frac{1}{\mu_y} & -\frac{\mu_x}{\mu_y} \end{array}\right]^t \\ &= \frac{\mu_x^2}{\mu_y^4}\left[\begin{array}{cc} -\frac{\mu_y}{\mu_x} & 1 \end{array}\right] \Sigma \left[\begin{array}{cc} -\frac{\mu_y}{\mu_x} & 1 \end{array}\right]^t \\ \text{Var}\ (s(x,y)) &= \frac{1}{\mu_y}\left[\begin{array}{cc} -\frac{\mu_y}{\mu_x} & 1 \end{array}\right] \Sigma \left[\begin{array}{cc} -\frac{\mu_y}{\mu_x} & 1 \end{array}\right]^t \end{aligned}$$

so

[1] In actual fact $\tilde{x}/\tilde{y} = 1/(c+u)$, not $1/c$, but since there is an estimate for the distribution of u from the regression of $\tilde{y}/\tilde{x}$, this approximation can be made more accurate. If the magnitude of the errors u/c are small, as is typically the case, this approximation will be good, as the variance term can be rewritten as $(u/c^2)(1/(1 + u/c))$. Otherwise the actual variation may need to be modelled for accurate results.

$$\begin{aligned}\frac{\text{SD }(r(x,y))}{r(\mu_x,\mu_y)} &= \frac{\mu_x}{\mu_y^2}\left\{\left[\begin{array}{cc} -\frac{\mu_y}{\mu_x} & 1\end{array}\right]\Sigma\left[\begin{array}{cc} -\frac{\mu_y}{\mu_x} & 1\end{array}\right]^t\right\}^{1/2}\left[\frac{\mu_x}{\mu_y}\right]^{-1} \\ &= \frac{1}{\mu_y}\left\{\left[\begin{array}{cc} -\frac{\mu_y}{\mu_x} & 1\end{array}\right]\Sigma\left[\begin{array}{cc} -\frac{\mu_y}{\mu_x} & 1\end{array}\right]^t\right\}^{1/2} \\ &= \left\{\left[\begin{array}{cc} -\frac{1}{\mu_x} & \frac{1}{\mu_y}\end{array}\right]\Sigma\left[\begin{array}{cc} -\frac{1}{\mu_x} & \frac{1}{\mu_y}\end{array}\right]^t\right\}^{1/2}\end{aligned}$$

$$\begin{aligned}\frac{\text{SD }(s(x,y))}{s(\mu_x,\mu_y)} &= \frac{1}{\mu_x}\left\{\left[\begin{array}{cc} -\frac{\mu_y}{\mu_x} & 1\end{array}\right]\Sigma\left[\begin{array}{cc} -\frac{\mu_y}{\mu_x} & 1\end{array}\right]^t\right\}^{1/2}\left[\frac{\mu_y}{\mu_x}\right]^{-1} \\ &= \frac{1}{\mu_y}\left\{\left[\begin{array}{cc} -\frac{\mu_y}{\mu_x} & 1\end{array}\right]\Sigma\left[\begin{array}{cc} -\frac{\mu_y}{\mu_x} & 1\end{array}\right]^t\right\}^{1/2} \\ &= \left\{\left[\begin{array}{cc} -\frac{1}{\mu_x} & \frac{1}{\mu_y}\end{array}\right]\Sigma\left[\begin{array}{cc} -\frac{1}{\mu_x} & \frac{1}{\mu_y}\end{array}\right]^t\right\}^{1/2}\end{aligned}$$

This shows that the coefficients of variation for the ratio and inverse ratio are asymptotically the same. So any observed difference in the coefficients of variation are due to speed of convergence to the asymptotic approximation and the validity of the assumption $\mathrm{E}[y/x]$ being close to $\mathrm{E}[y]/\mathrm{E}[x]$. To illustrate how this might work in practice, we turn to some simple simulations.

We can use the following model (a variant of a common errors in variables setup) to illustrate some of the convergence issues. Define

$$\begin{aligned} y &= (\tilde{y}+v) \\ x &= (\tilde{x}+u) \\ \mathrm{cv}(y/x) &= \mathrm{sd}(y/x)/\mathrm{mean}(y/x) \\ \mathrm{cv}(x/y) &= \mathrm{sd}(x/y)/\mathrm{mean}(x/y), \end{aligned}$$

where $\tilde{y}$ and $\tilde{x}$ are assumed to have normal distributions with means 300 and 200, respectively, and v and u are error terms with zero means. We run simulations of 10,000 observations, perturbing the standard deviations of these and display the results in Table S1.

Increased variation in u and v increases the CV's of x and y, obviously. But increased variation that follows a ratiometric pattern does not necessarily increase the CV's of the ratios, as is now shown.

We model explicitly ratiometric behavior (in the sense of Schnute), by simulating 10,000 observations where the mean of x is 200, with standard deviation 20, and 10,000 observations where the mean of x is 300, also with standard deviation 20. Similarly for y, 10,000 observations are simulated with a mean of 400 and 10,000 observations with a mean of 600. Thus the ratio of y/x is equal to 1.5 for each set of observations, but the standard deviations of x and y will be very large, since the difference of the two means of x is 100 and the difference of the two means of y is 150. The results are display in Table S2.

The standard deviations of x and y are as expected and equal to what was described above. As we would expect from the Schnute model (which these simulation parameters fit), though, the CV's are low, lower, actually, than any in Table S1. These simulations would thus seem to fit the analytic results derived above – a ratiometric relationship implies CV's closer to one another than would be expected notwithstanding large variation in the marginal distributions.

Statistical test of $CV(FPKM)$ groupings

In the paper an analysis of gene variation (as defined by the $CV(FPKM)$ of the KEGG pathway genes is presented. This analysis is performed by dividing the pathways into four groups: 1) pathways recovered by the RA approach with a wide margin compared to PE or MI; 2) pathways recovered similarly by all methods, but slightly better by the RA analysis; 3) pathways for which the three methods identify an approximately equal number of genes; and 4) pathways better recovered by PE and MI. It is concluded that the values of $CV(FPKM)$ are lowest for pathways in the first group, and highest for pathways that are in the fourth group. We now provide evidence that the first and fourth groups are different than all the other groups and that the second and third groups are statistically indistinguishable.

To provide a statistical framework for this statement, we turn to linear regression and regress the $CV(FPKM)$ of the gene on dummy variables, coded as follows: $c1$ is set to one if the gene is in group 1, 0 otherwise, $c2$ is set to one if the gene in group 2, 0 otherwise, $c3$ is set to one if the gene is in group 3, 0 otherwise, and $c4$ is set to one if the gene

is in group 4, 0 otherwise (the constant term is of course not included in the estimation since $c1 + c2 + c3 + c4 = 1$, by definition). The regression is given in Table S3.

All of these coefficients are highly significantly different than zero. The interpretation of this regression is that the average effect of being in a group is the coefficient of the group. Thus the first column of Table S3 is the average $CV(FPKM)$ of each group. We are interested in the null hypotheses that all groups have the same average $CV(FPKM)$, and we do this by testing whether the coefficient of one group is statistically different from the coefficient of another group, for all six possible combinations (group 1 compared to 2, 3, and 4, group 2 compared to 3 and 4, and group 3 compared to 4).

To test whether two groups have the same $CV(FPKM)$, we need to divide by the standard deviation of the difference of those two coefficients to obtain a t statistic. Since both coefficients are random quantities and have a covariance, that standard deviation is the square root of the sum of the individual variances for each coefficient minus twice the covariance of the two coefficients. All of these variance/covariance quantities can be obtain from the covariance matrix of the linear regression. These t-values can be displayed in matrix form, as they are in Table S4 (here Second Group is the t-value of the coefficient of that group being subtracted from the coefficient First Group).

These t-values are all very high, except for the t-statistics representing the difference between groups 2 and 3. Since we have a sizeable number of observations for each group, a normal approximation to the t statistic is valid, the t-values for the difference between groups 1 and the remaining groups are all significant at well below the .001 level, and for between group 4 and the others groups, at below the .005 level. Groups 2 and 3, with a t-value of 0.54, are not significantly different at the .05 level (given the magnitude of these t-values, it is clear that these conclusions obtain even if a Bonferroni type procedure is applied). In particular, our statement that group 1 has a smaller $CV(FPKM)$ than the other groups and group 4 has a larger $CV(FPKM)$ than the other groups is statistically supported.

Supplementary References

Bickel PJ, Doksum KA. 1997. Mathematical Statistics. *San Francisco, CA: Holden-Day, Inc.*

Supplementary Tables

Table S1: CV's as function of input variation

sd(x)	sd(y)	sd(u)	sd(v)	$cv1$	$cv2$
20	20	.01	.01	0.1225757	0.120245
20	20	.1	.1	0.1244791	0.1219522
20	20	1	1	0.1243477	0.1223783
20	20	10	10	0.1379601	0.1351505
20	20	20	20	0.1756124	0.1696806
20	20	40	40	0.3236766	0.2829277
40	40	.1	.1	0.2754183	0.2467305
40	40	.01	.01	0.2759232	0.249387

Table S2: CV's as function of input variation, Schnute ratio

sd(x)	sd(y)	sd(u)	sd(v)	$cv1$	$cv2$
100.99	150.79	10	10	0.1097509	0.1076288

Table S3: Regression of $CV(FPKM)$ on group membership dummy variables

	Estimate	Std. Error	t value	Pr($>|t|$)
$c1$	0.195424	0.004536	43.09	$< 2e-16$
$c2$	0.219751	0.003410	64.44	$< 2e-16$
$c3$	0.222762	0.004308	51.71	$< 2e-16$
$c4$	0.242154	0.005370	45.09	$< 2e-16$

Table S4: t values for difference of $CV(FPKM)$ for groups

		First group			
		1	2	3	4
Second	1	.	4.28	4.36	6.65
Group	2	.	.	0.54	3.52
	3	.	.	.	2.81
	4	.	.	.	.

Table S5: Vertex sizes $|V(G)|$ with corresponding stringency cut offs and gene counts for each method.

	Ratiometric		Pearson correlation		Mutual information	
i	$\|V(G)\|$	CV	$\|V(G)\|$	R^2	$\|V(G)\|$	I
1	22	0.08	28	0.89	22	1.02
2	182	0.09	166	0.85	177	0.87
3	631	0.1	623	0.8	618	0.76
4	1,359	0.11	1,303	0.75	1,363	0.66
5	2,220	0.12	2,217	0.7	2,226	0.59
6	3,116	0.13	3,184	0.65	3,124	0.53
7	4,300	0.145	4,234	0.6	4,214	0.47
8	5,567	0.165	5,539	0.54	5,517	0.41
9	6,950	0.205	6,863	0.47	6,905	0.35
10	8,012	0.265	7,993	0.4	8,022	0.3
11	9,563	1.0	9,565	0.22	9,580	0.19

Table S6: Edge sizes $|E(G)|$ with corresponding stringency cut offs and gene pair relationship counts for each method.

	Ratiometric		Pearson correlation		Mutual information	
i	$\|E(G)\|$	CV	$\|E(G)\|$	R^2	$\|E(G)\|$	I
1	56	0.085	68	0.87	61	0.95
2	411	0.095	484	0.83	462	0.85
3	4581	0.110	4,919	0.76	4545	0.72
4	8618	0.115	8,043	0.74	8384	0.68
5	42,843	0.130	45,923	0.65	43,474	0.56
6	99,112	0.140	95,861	0.60	104,570	0.49
7	467,497	0.165	476,435	0.46	505,308	0.36
8	1,088,031	0.185	1,089,421	0.37	1,069,168	0.30
9	2,006,347	0.205	1,963,503	0.30	2,072,871	0.25
10	4,943,161	0.255	4,838,371	0.19	4,997,296	0.19
11	1.01×10^7	0.395	1.07×10^7	0.10	1.01×10^7	0.15

Supplementary Figures

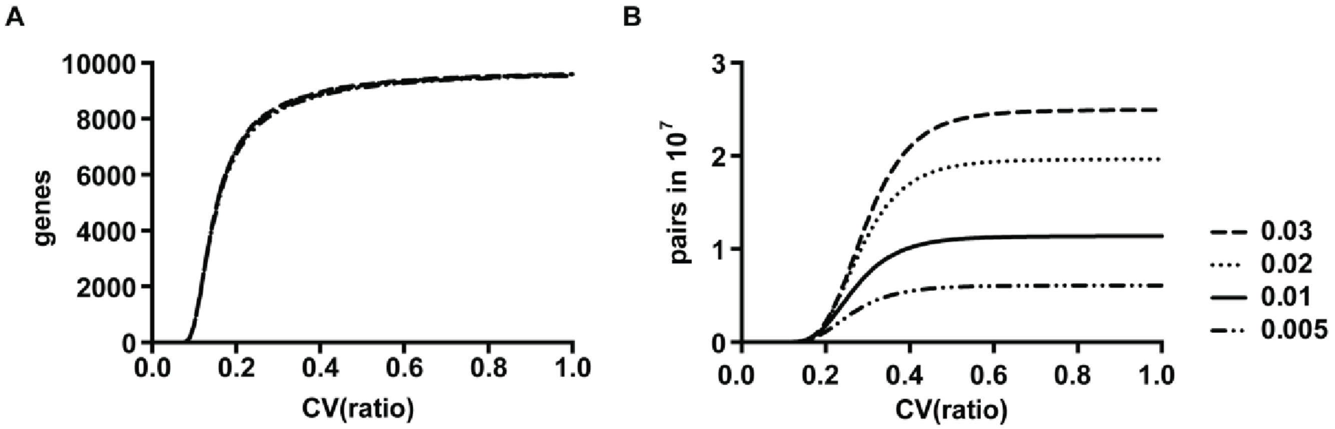

Figure S1: Connectivity trends in gene graphs built by the RA method using different Δ_{CV} cutoffs. Analyzing the effect the Δ_{CV}-cutoff has on the inclusion rate per stringency level by plotting **(A)** the number of unique genes and **(B)** the number of gene pairs found at every CV level ranging from 0 to 1. The Δ_{CV} has no noticeable effect on the rate of gene inclusion to the cluster. The effect can be seen in the number of gene pairs found, though first after ∼50% of the genes are already included. As the most gene expression analyses focus on the top ranked interactions, the effect of Δ_{CV} is minimal for this dataset.

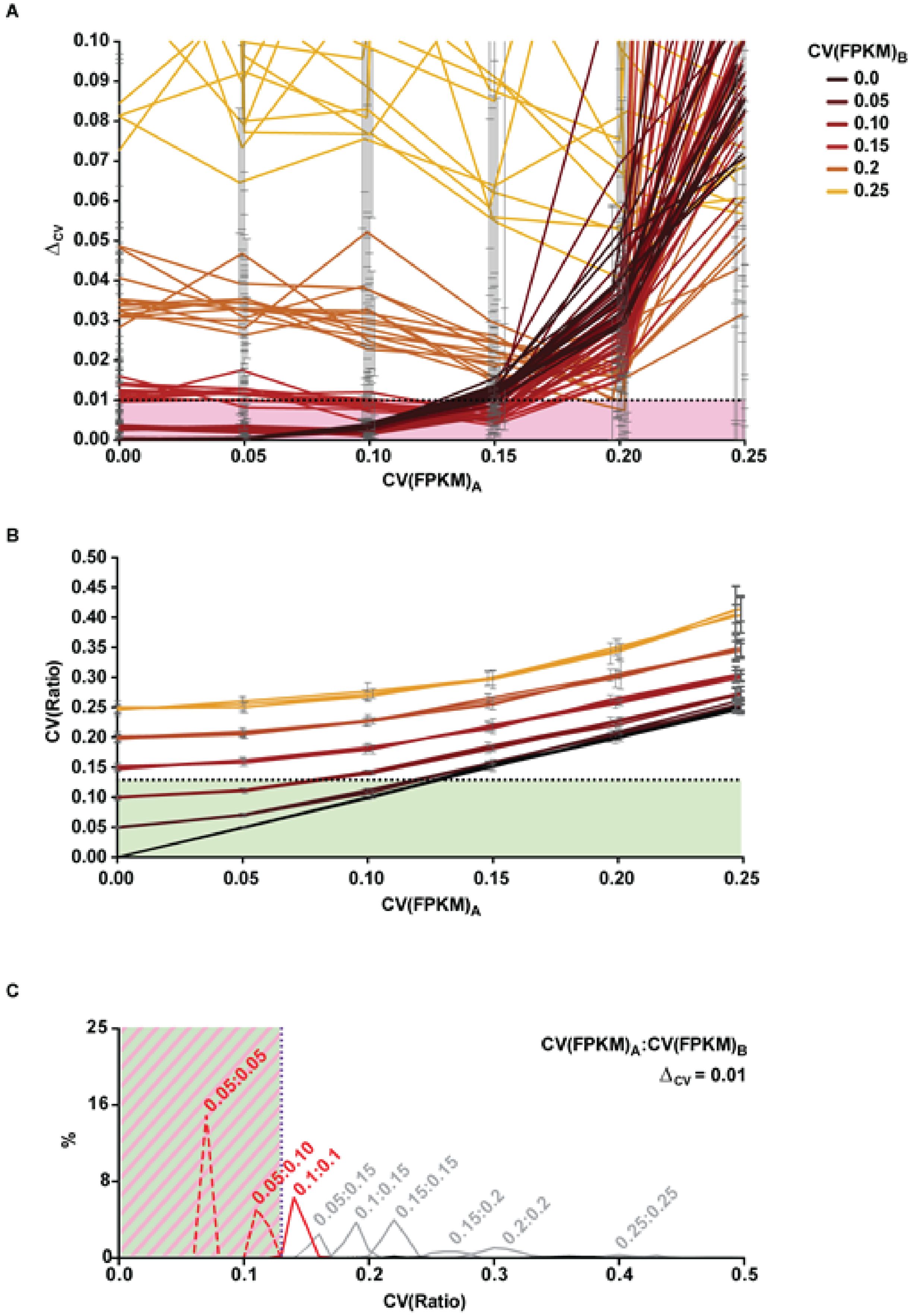
A
CV(FPKM)B
0.0
0.05
0.10
0.15
0.2
0.25
ΔCV
CV(FPKM)A
B
CV(Ratio)
CV(FPKM)A
C
CV(FPKM)A:CV(FPKM)B
ΔCV = 0.01
%
0.05:0.05
0.05:0.10
0.1:0.1
0.05:0.15
0.1:0.15
0.15:0.15
0.15:0.2
0.2:0.2
0.25:0.25
CV(Ratio)

Figure S2 ***(preceding page)*****: Simulation assessing the degree to which two independently expressed genes produce false positives**. The expression levels of two genes, A and B, were generated independently of each other according to $A \sim \mathcal{N}(\mu, \sigma)$ and $B \sim \mathcal{N}(\mu, \sigma)$, $\mu = [1, 2, 5, 10, 20, 50, 100, 500, 1000]$ and $\sigma = p\mu/100$ where $p = [0, 5, 10, 15, 20, 25]$. Each run included 10 iterations and each iteration had an $n = 462$ (the number was chosen to mirror the dataset size used in chapter 3). The runs are colored according to $CV(FPKM)_B$. **(A)** Δ_{CV} versus $CV(FPKM)_A$ when varying $CV(FPKM)_B$, demonstrating that Δ_{CV} both increases in value and fluctuation as the $CV(FPKM)$ increases. Pink area indicates the Δ_{CV}-cut-off used in chapter 3. **(B)** $CV(Ratio)$ versus $CV(FPKM)_A$ when varying $CV(FPKM)_B$, demonstrating that $CV(Ratio)$ increases as the $CV(FPKM)$ increases. Green area indicates the $CV(Ratio)$-range plotted in the KEGG-analysis in chapter 3. **(C)** The percentage of accepted gene pairs for the different $CV(FPKM)$-values tested (given over each peak as the $CV(FPKM)_A$:$CV(FPKM)_B$) at $\Delta_{CV} = 0.01$. Only curves demonstrating at least one peak $\geq 1\%$ are plotted. The striped area indicates the chosen cut-offs used for the B-cell dataset. Red color indicate $CV(FPKM)$, potentially in the risk zone of producing false positives. The dashed red lines are the subgroup of the latter exhibiting a $CV(FPKM)$ not present in the B-cell dataset. The solid red line is the only case where $CV(FPKM)$s presents a possible risk of introducing false positives in the B-cell dataset. Out of 2430 possible gene pairs with 0.1:0.1, only five were reported by RA, i.e. an error rate of 0.2%. The gray curves are outside the cut-off levels.

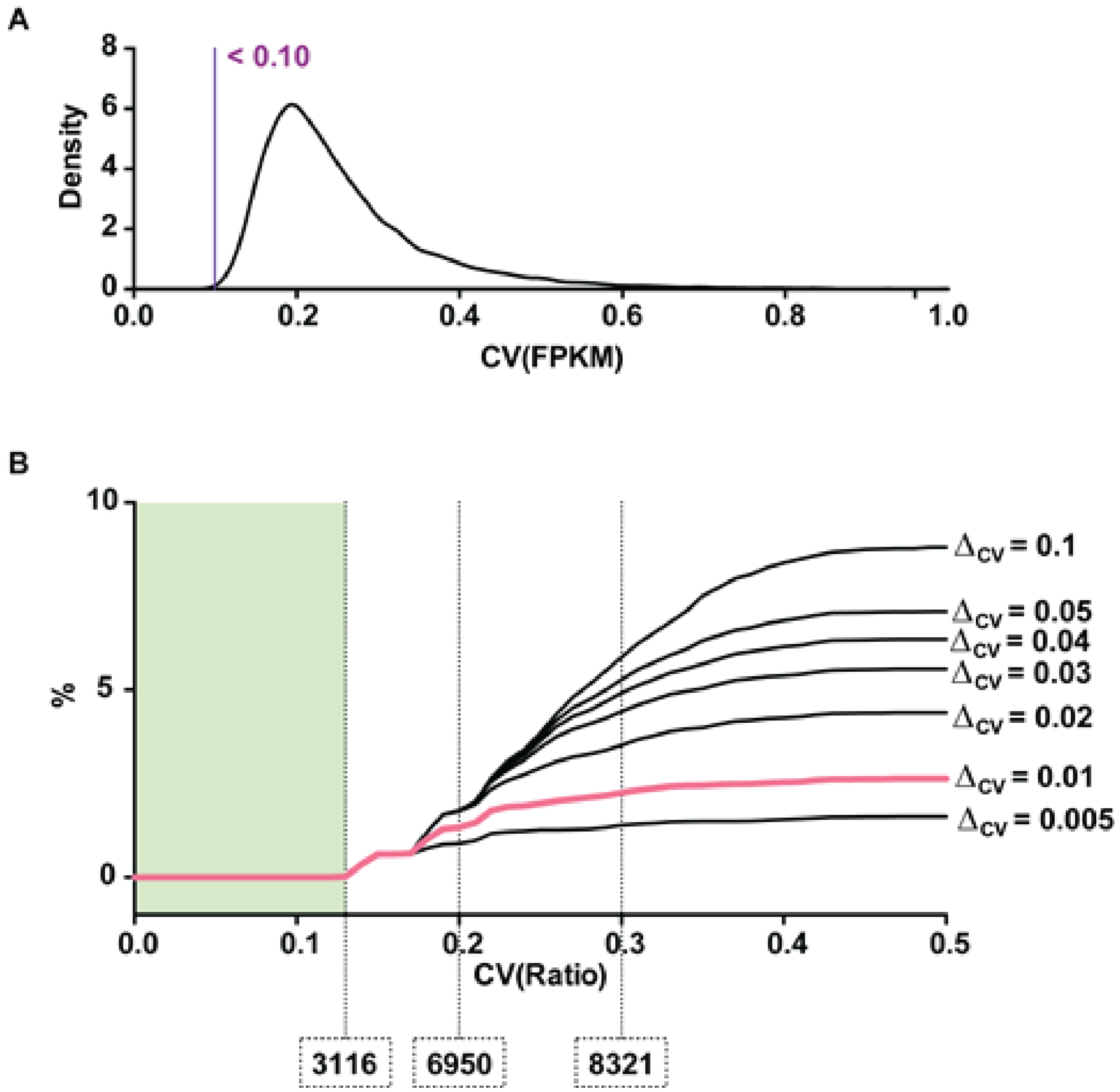

Figure S3: Estimating the number of false positives in the B-cell bulk RNA-seq data. **(A)** Distribution of $CV(FPKM)$ for all genes in the dataset. A $CV(FPKM) \leq 0.10$ is indicated (purple line), which in the B-cell dataset includes no genes. **(B)** The percentage of accepted gene pairs when simulating the expression of two independently generated genes, for varying Δ_{CV} levels. The gene counts at $CV(Ratio)$ levels of 0.13, 0.2 and 0.3 are shown in dotted boxes. To mimic the real-life dataset the simulation results were adjusted to only include genes with $CV(FPKM) \geq 0.1$, i.e. [0.1, 0.15, 0.20, 0.25]. The chosen Δ_{CV} of 0.01 is colored in pink and the $CV(Ratio)$ range, 0 to 0.13, analyzed in the KEGG-analysis is marked in green. Such settings minimizes the number of false positives reported to ~0.015%.

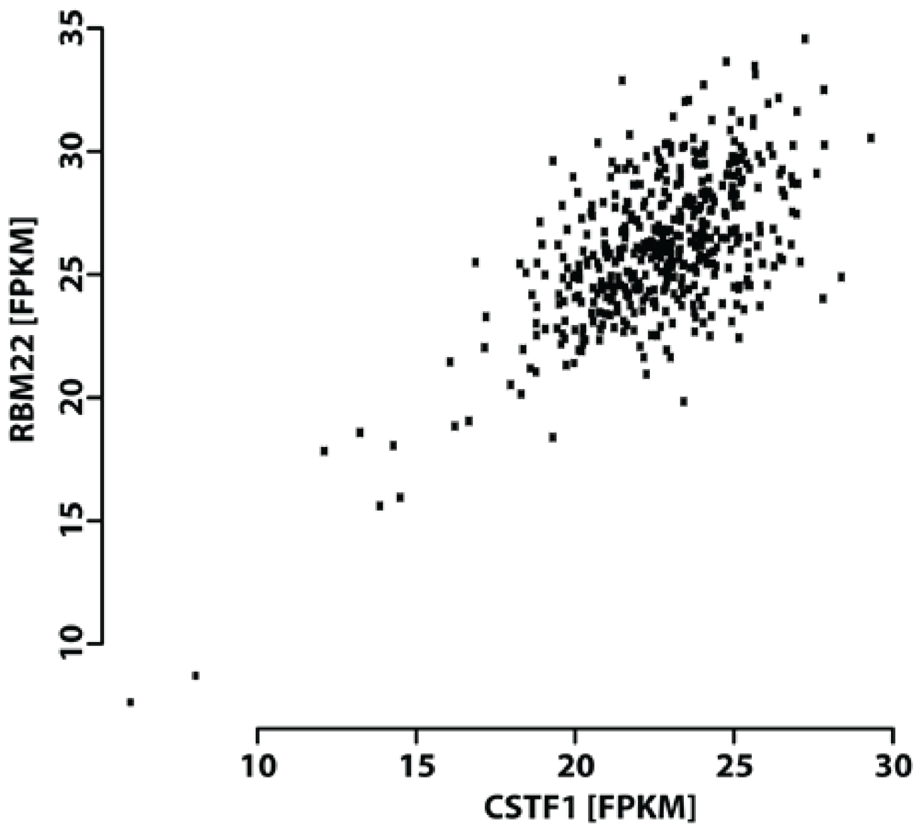

Figure S4: An example of ratiometric behavior with low R^2. The gene pair (*CSTF1*, *RBM22*) has a relatively low $R^2 = .4$, but also fits the ratiometric definition, with $\Delta_{CV} = 0.001$ and $CV(A/B) = 0.10$, $CV(B/A) = 0.10$.

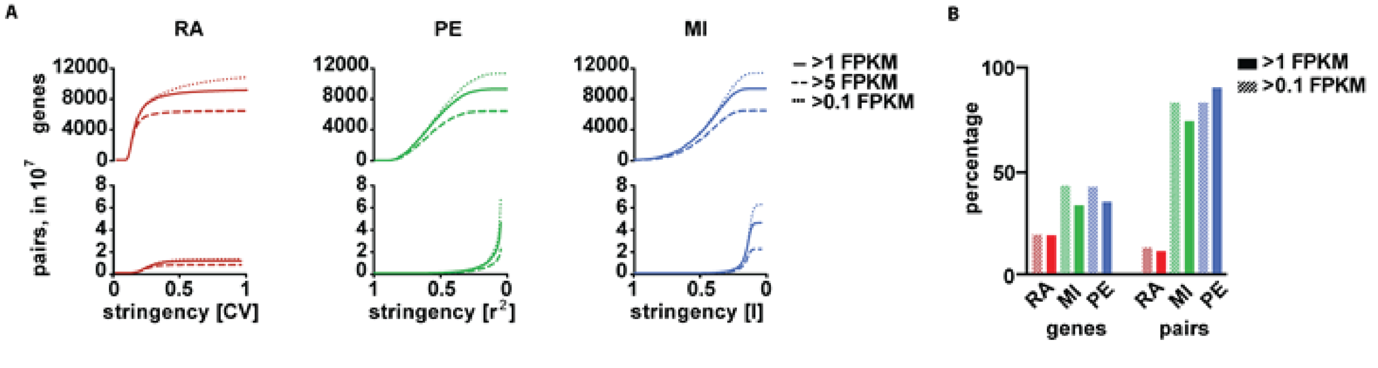

Figure S5: Gene set selection. The gene set analyzed includes all genes with FPKM ≥ 1 for at least 95% of the samples. To explore the effect of the FPKM cutoff used, we analyzed cutoff of 0.1 and 5 FPKM. **(A)** Each set was analyzed according to the RA, PE and MI methods, reporting how many gene pairs and genes are found at each stringency level, RA : $[0 \rightarrow 1]$, PE : $[1 \rightarrow 0]$ and MI : $[2 \rightarrow 0]$. B) An example of the differences between the gene sets for the 3 methods. The difference (in percentage) of gene pairs and genes included for the gene sets ≥ 1 FPKM and ≥ 0.1 FPKM, at the same stringency level that results in $\sim$5000 incorporated genes for the gene set ≥ 5 FPKM. The RA method shows a lower degree of variation across the three gene sets than PE and MI.

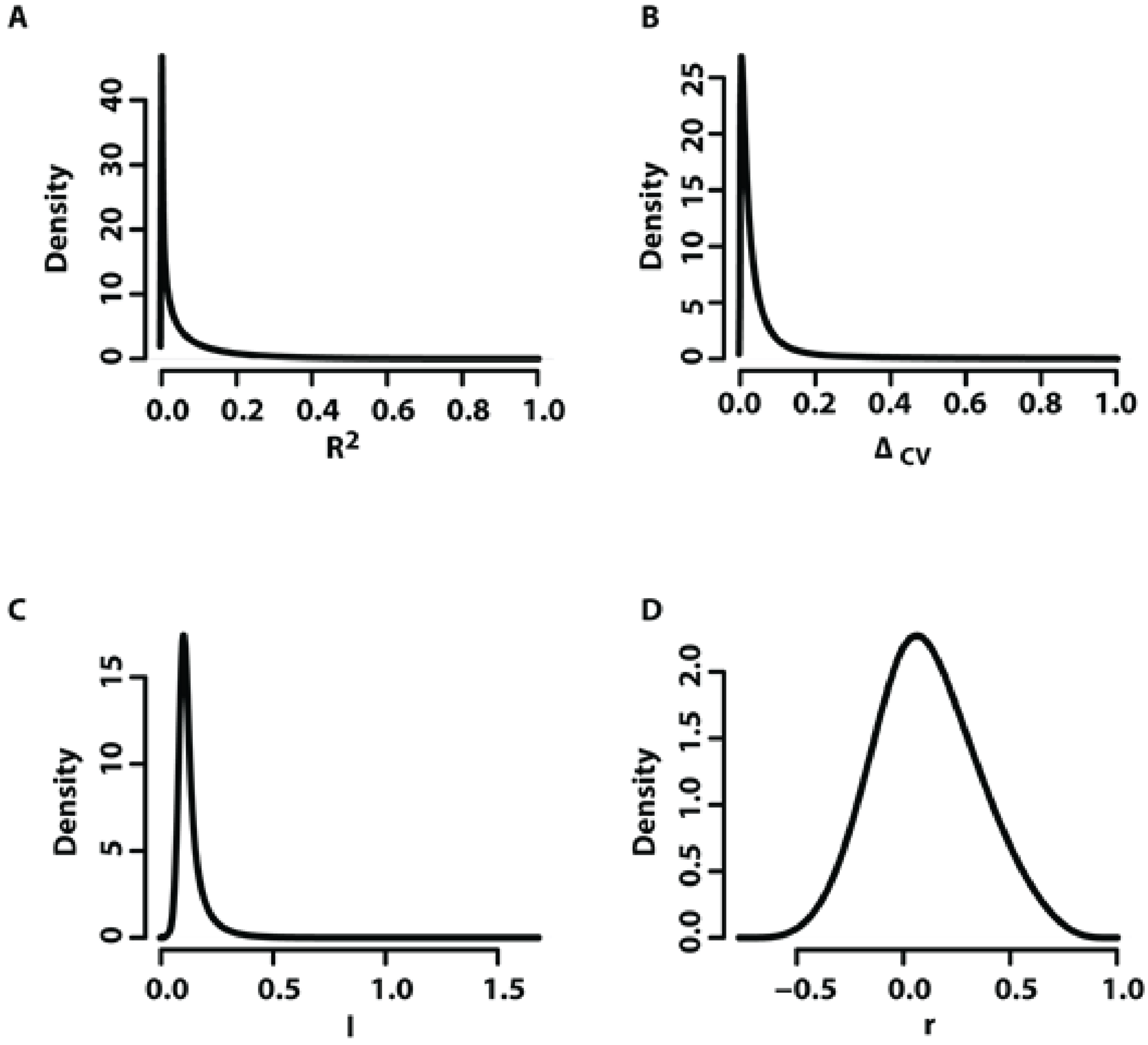

Figure S6: The distributions of the three measures of association. The distributions of the three measures of association (R^2, MI and Δ_{CV}) are plotted, along with the sample Pearson correlation coefficient (r). The overall distributions of R^2, I and Δ_{CV} are relatively similar in appearance, though as demonstrated in the main text, R^2 and MI are highly correlated with one another and Δ_{CV} is not correlated with either.

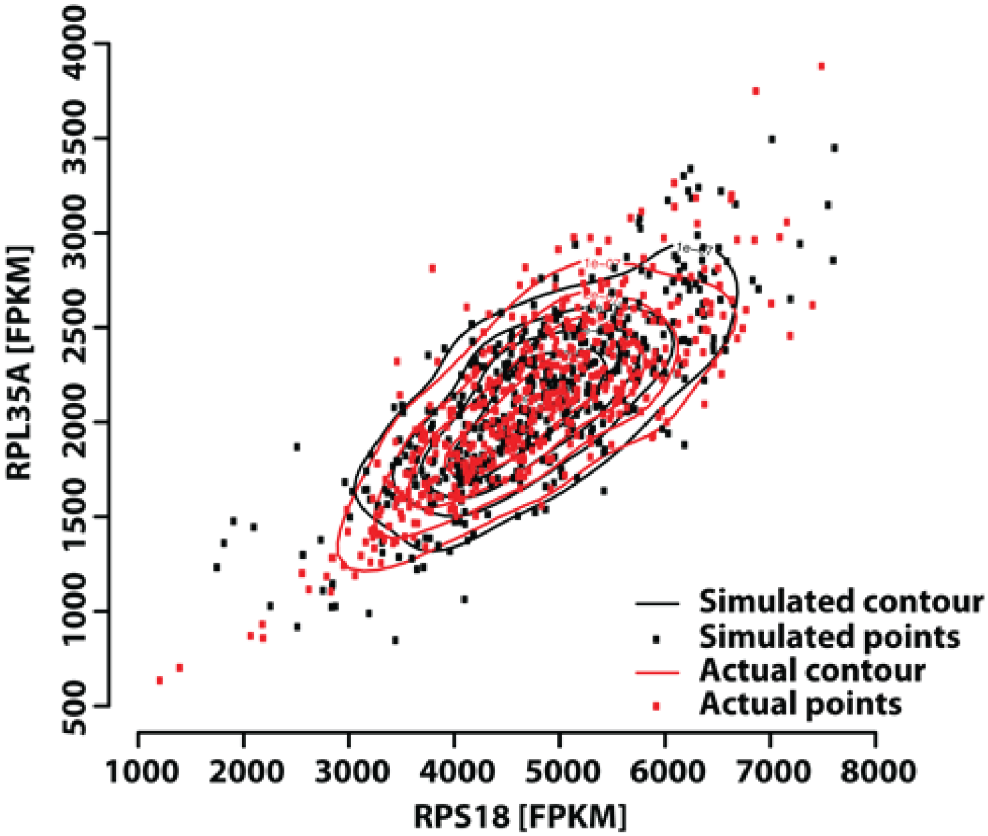

Figure S7: The ribosomal protein gene pair *RPS18* and *RPL35A* display a typical ratiometric profile. Observations from primary data are plotted for *RPS18* versus *RPL35A* (red points), and contours are shown (red line). Under the working assumption that the joint distribution of *RPS18* and *RPL35A* is bivariate normal, the same number of simulated observations from this distribution is plotted (black points) with the corresponding contour (black line). Both *RPS18* and *RPL35A* are univariate normal at the .05 level by the Kolmogorov-Smirnov test, but their joint distribution is not bivariately normal (by the `HZ.test` in the R package `MVN`). The contours for data (red) tend to fall within those for the simulated distribution. This form of ratiometric behavior can therefore occur through reduced variation by collapsing to $A/B = c$. As mentioned in the text, a statistical approach would consist of testing whether the variation of the joint distribution, given the marginal distributions, is statistically different than what was expected, as is the case here (two univariate normals are typically assumed to be bivariately normally distributed, though they need not be, as shown in this example).

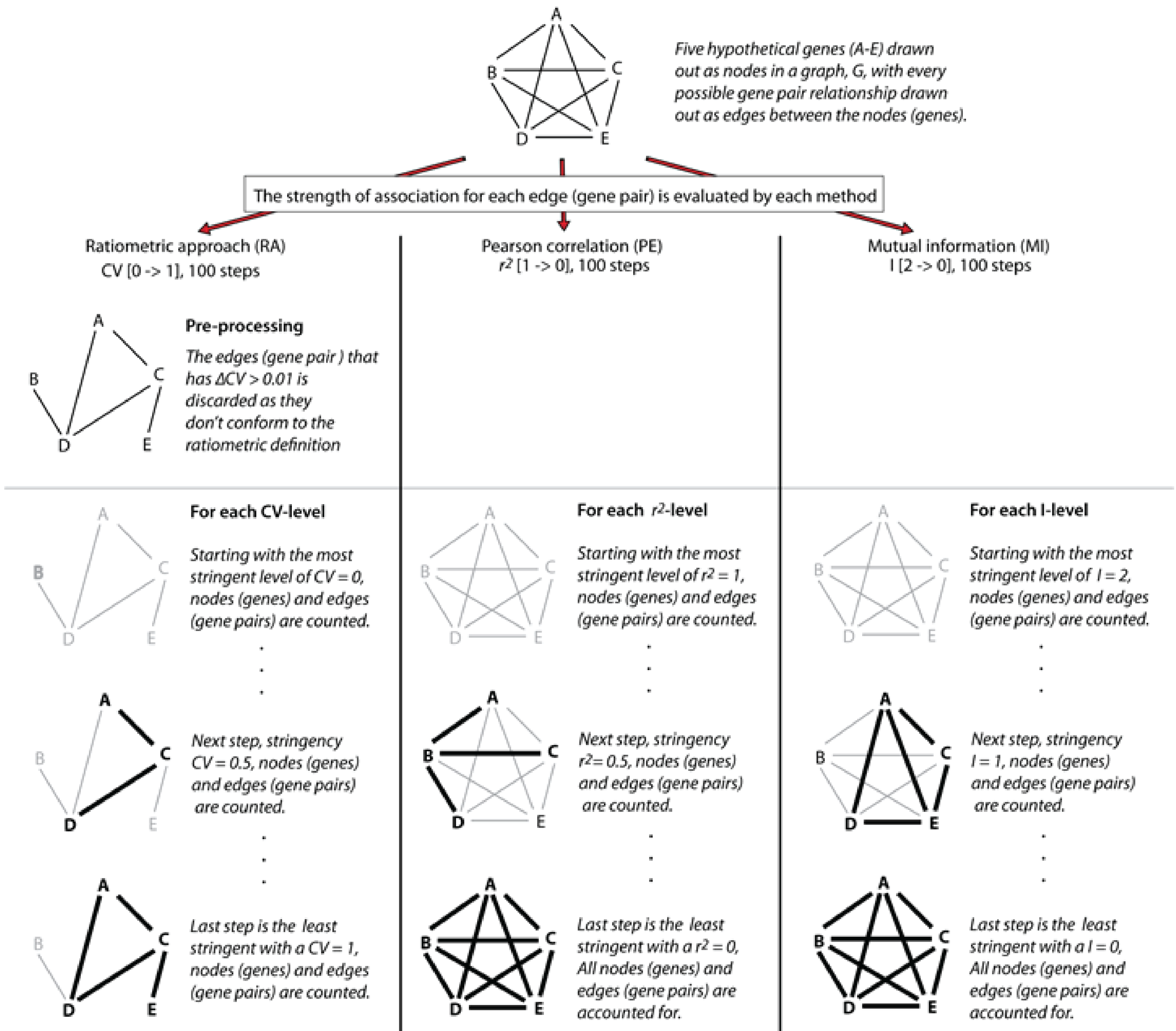

stringnecy [CV]	# nodes (genes)	# edges (gene pairs)
0	0	0
. . .	. . .	. . .
0.5	3	2
. . .	. . .	. . .
1.0	4	4

stringnecy [r^2]	# nodes (genes)	# edges (gene pairs)
1	0	0
. . .	. . .	. . .
0.5	4	3
. . .	. . .	. . .
0	5	10

stringnecy [I]	# nodes (genes)	# edges (gene pairs)
2	0	0
. . .	. . .	. . .
1	4	5
. . .	. . .	. . .
0	5	10

Figure S8 ***(preceding page)*****: Schematic example illustrating how the methods process a hypothetical dataset of 5 genes.** Using five fictive genes, which create 10 possible gene pair combinations, we can construct a graph where the genes are nodes and the pairwise gene interactions are edges. The processing of the expression data by Pearson correlation, PE, and mutual information, MI, is relatively straightforward. By stepwise decreasing the stringency level, for PE, R^2 and MI, from most stringent, $R^2 = 1$ and $MI = 2$, to least stringent, $R^2 = 0$ and $MI = 0$, and at each step count the number of nodes and edges (thicker lines), a continuous reporting of how the graph increases is produced. Note that for both PE and MI all genes and gene pair combinations will be counted at the lowest stringency level. For the ratiometric method, RA, there is a pre-processing step to select for gene pairs that exhibit a ratiometric expression pattern, $\Delta_{CV} \leq$ the chosen cut off value. Only these relationships are included in the analysis. Then, the same approach is applied, where the stringency is measured in CV. The most stringent level is $CV = 0$ and the least stringent level used is $CV = 1$. Notice that due to the pre-processing step and the fact that CV goes to infinite, there is a possibility that not all genes and all gene pairs are included in the analysis.

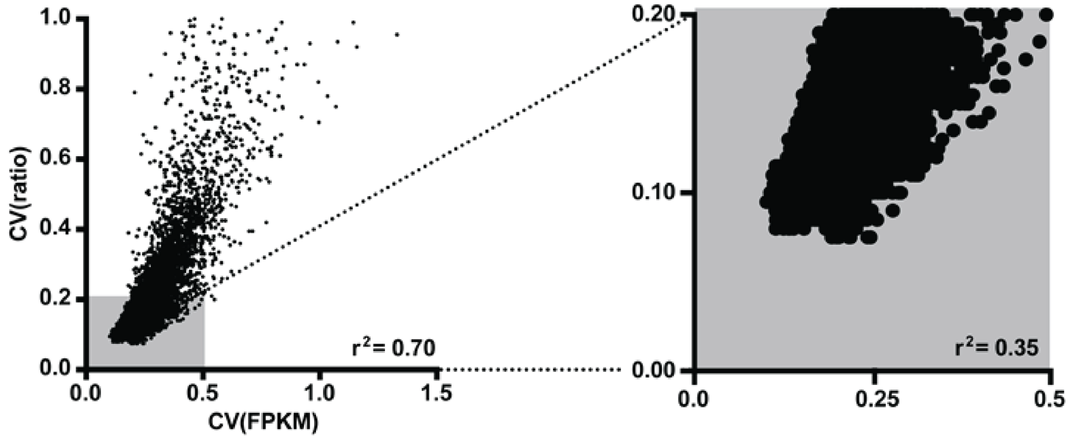

Figure S9: $CV(FPKM)$ **versus** $CV(ratio)$ **for the 9563 genes found by the RA method.** Shown is the plott of the $CV(FPKM)$ against the $CV(ratio)$ from the most stable ratio per gene. For the entire gene set $CV(FPKM)$ correlates with $CV(ratio)$, but not among the top 7000 RA-ranked genes (see zoomed graph, $CV(FPKM) < 0.5$, and $CV(ratio) < 0.2$). Hence for the majority of the genes and especially the top RA-ranked genes, the degree of gene expression dispersion does not predict the RA-ranking order.

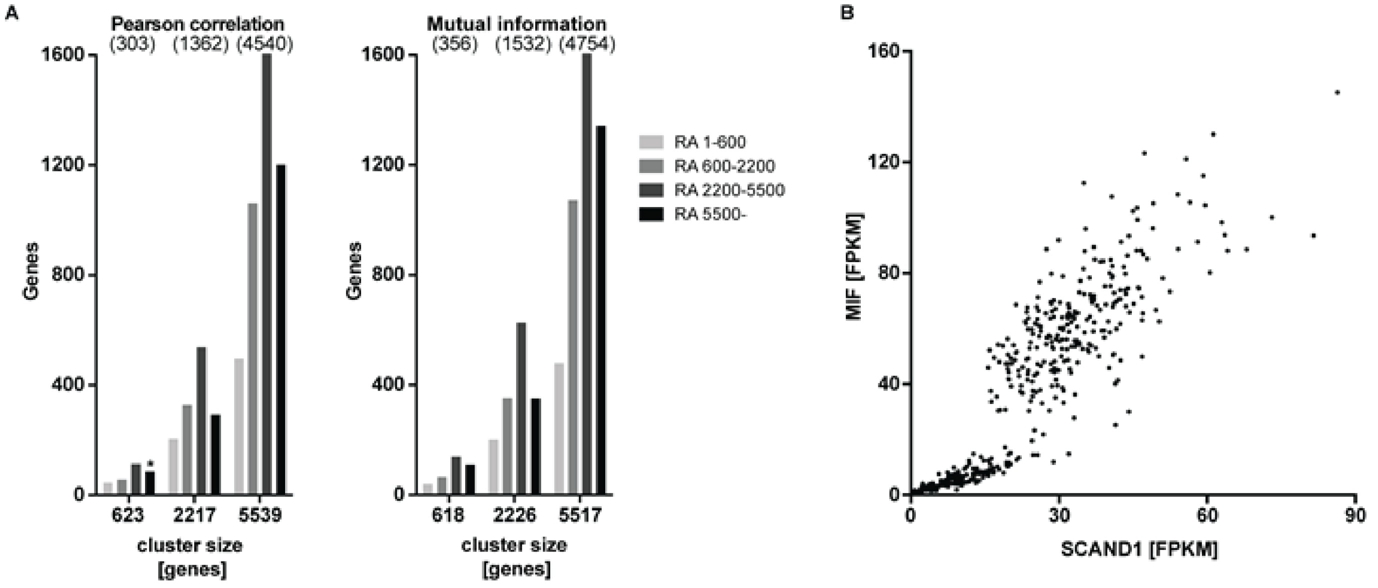

C

GO term	p-value	Bonferroni corrected
Regulation of apoptosis	$1.3 * 10^{-3}$	0.604
Reg. of prog. cell death	$1.4 * 10^{-3}$	0.632
Reg. of cell death	$1.5 * 10^{-3}$	0.642
Positive reg. of apoptosis	$7.7 * 10^{-3}$	0.996
Positive reg. of prog. cell death	$8.0 * 10^{-3}$	0.996
Positive reg. of cell death	$8.2 * 10^{-3}$	0.997
Reg. of transcription	0.012	1.0
Phosphorylation	0.015	1
Reg. of phosphorylation	0.04	1
Reg. of caspase activity	0.04	1

Figure S10: Analysis of gene pairs highly ranked by PE and MI but rejected by RA. (A) For 3 cluster sizes (∼600, ∼2200, ∼5500), the number of genes involved in a gene pair relationship not accepted by the Δ_{CV} cut off (total number given in parenthesis above bars) is binned into one of four groups (1-600, 600-2200, 2200-5500, 5500-) according to which RA cluster size it makes its first appearance. **(B)** An example of a gene pair (*SCAND1* and *MIF*) which exhibit a two-subgroup pattern. Roughly 40% of the gene pairs in the top PE and MI clusters found in RA_{5500-} have such behavior. **(C)** For PE, the first 10 GO terms (biological processes) for the gene group in the top cluster size (623), found in the largest cluster size (5500-) in the RA cluster, column marked with * in (A). Gene pairs not accepted by the RA method and found in the top cluster in both the PE and the MI, contains genes that to the most part, are low ranked in the RA method. This gene group is enriched in GO terms involved in cell death and its regulation.

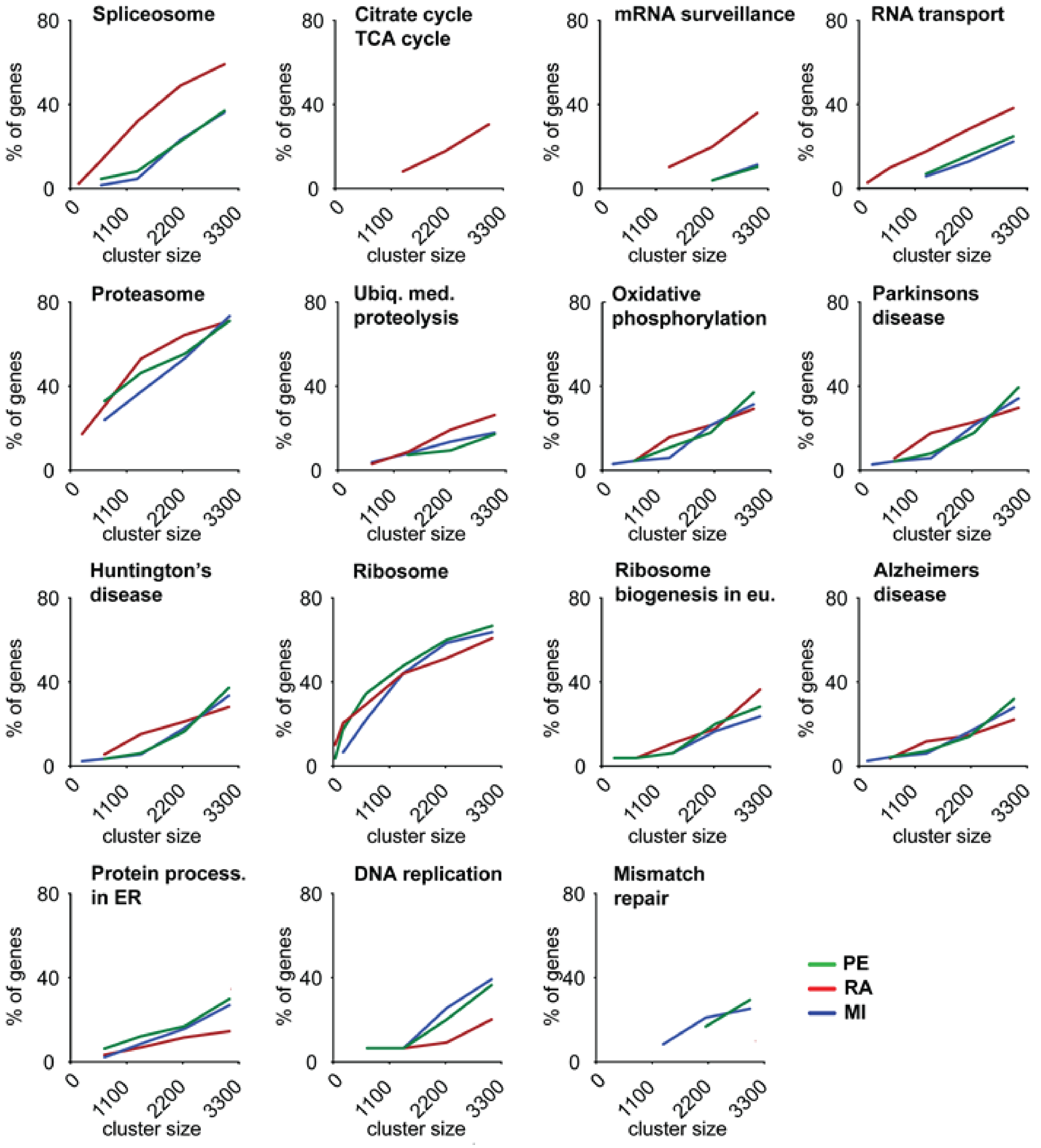

Figure S11: KEGG pathway enrichment analysis, gene coverage. The percentage of genes in a pathway discovered is plotted against the increasing graph size. The RA method, red, MI, blue, PE, green. A gene is included if it is in at least one relationship with another gene from the same pathway. The same pathways as in figure 5 are plotted. Dashed lines are perturbed runs simulating the real pathway.

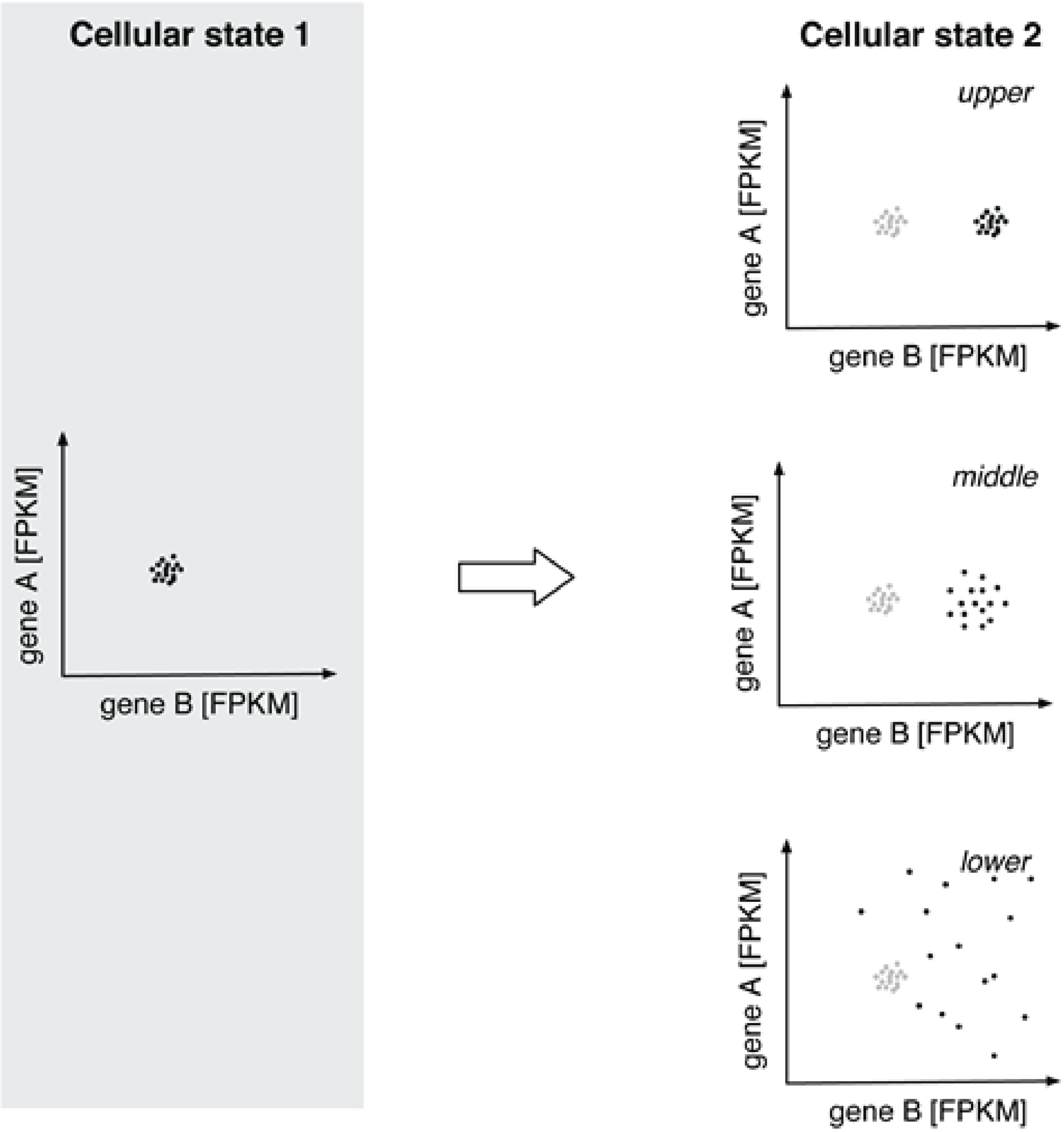

Figure S12: Schematics of unique detection by RA. The gene pair relationship in cellular state 1 is only reported by the RA method compared to PE and MI, as the latter cannot detect a gene pair combination with such low expression dispersion rate. When information from the second cellular state is added, here three different scenarios are given, the gene pair relationship remain undetected by PE and MI, but according to the RA method different outcomes would be reported. The upper graph indicates that the gene pair relationship has remained stable between the two cellular states but it has shifted its ratiometric value from 1:1 to 1:2. The middle graph also exhibits the shift of ratiometric value but additionally it has loosened the stability of the ratio. The last, lower, graph, displays a scenario when the gene pair relationship has dissolved in the second cellular state. Note that these three scenarios show changes in a gene pair relationship that could be vital for understanding the molecular changes occurring in the two different states and moreover, they are undetected by the commonly used methods PE and MI.
